# Androgen‐repressed lncRNA LINC01126 drives castration‐resistant prostate cancer by regulating the switch between O‐GlcNAcylation and phosphorylation of androgen receptor

**DOI:** 10.1002/ctm2.1531

**Published:** 2024-01-12

**Authors:** Yi Cai, Minfeng Chen, Yuchen Gong, Guyu Tang, Zhiwei Shu, Jiaxian Chen, Hengfeng Zhou, Yao He, Zhi Long, Yu Gan

**Affiliations:** ^1^ Department of Urology Disorders of Prostate Cancer Multidisciplinary Team National Clinical Research Center for Geriatric Disorders Xiangya Hospital Central South University Changsha Hunan P.R. China; ^2^ Andrology Center Department of Urology The Third Xiangya Hospital Central South University Changsha Hunan P.R. China

**Keywords:** androgen receptor, castration‐resistant prostate cancer, long non‐coding RNA, post‐translational modifications

## Abstract

**Background:**

Prostate cancer (PCa) initially shows satisfactory response to therapies targeting the androgen receptor (AR). However, progression to a castration‐resistant stage indicates poor prognosis in PCa patients. AR signalling still plays a central role in most castration‐resistant prostate cancers (CRPC). Therefore, unveiling the mechanisms of AR reactivation under androgen‐deprived conditions is imperative to discover novel therapeutic targets for CRPC.

**Methods:**

Using an integrative analysis of the transcriptomics of three independent PCa cohorts and a published landscape of AR‐regulated long non‐coding RNA (lncRNA), lncRNA LINC01126 was selected as a candidate gene that could drive CRPC progression for further study. Quantitative reverse transcription polymerase chain reaction, in situ hybridisation (ISH) and fluorescent ISH were performed to detect LINC01126 in PCa tissues and cells. The functional role and mechanism of LINC01126 were further investigated using in vitro and in vivo gain and loss of function assays.

**Results:**

LINC01126, identified as an AR‐repressed lncRNA, was significantly upregulated after AR‐targeted therapies. In addition, we found that LINC01126 was upregulated in CRPC and was associated with poor prognosis. We also proved that LINC01126 stabilised AR protein and enhanced AR nuclear translocation and transactivation by promoting the transition from O‐GlcNAcylation at threonine 80 to phosphorylation at serine 81 (S81) within the AR protein. Mechanism analysis revealed that LINC01126 facilitates the interaction of CDK9 with AR and impedes the binding of O‐linked N‐acetylglucosamine (O‐GlcNAc) transferase to AR. Consequently, LINC01126 expression was sufficient to activate AR signalling without androgen. LINC01126 overexpression increased, whereas LINC01126 knockdown decreased castration resistance traits in PCa cells in vitro and in vivo. Furthermore, our data showed that LINC01126‐targeting antisense oligonucleotides (ASO) substantially inhibited CRPC cells in vitro.

**Conclusions:**

Our research expands the functions of AR‐regulated lncRNA in sustaining androgen‐independent AR activity and promoting CRPC progression and reveals that LINC01126 may be a new therapeutic target for PCa.

## Graphical abstract

LINC01126 is highly expressed in castration‐resistant prostate cancer and is correlated with poor prognosis.

AR‐targeted therapies upregulate LINC01126 expression through relieving its transcriptional repression by AR.

LINC01126 promotes the transition from O‐GlcNAcylation at threonine 80 to phosphorylation at S81 within the AR protein.

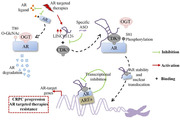

## 1 INTRODUCTION

Prostate cancer (PCa) ranks the second most prevalent cancer and the fifth leading cause of cancer‐related death in men globally.^1^, ^2^ Since the androgen receptor (AR) plays a pivotal role in the development and progression of PCa, androgen‐deprivation therapy (ADT) targeting AR signalling has become the mainstay systemic treatment for patients with locally advanced, biochemically recurrent and metastatic disease.^2^ Although most tumours initially respond well to ADT, almost all inevitably relapse with castration‐resistant PCa (CRPC), which is generally considered a fatal disease with a poor prognosis.^2^, ^3^ A major explanation of CRPC pathogenesis is the recovery of AR signalling independent of ADT through multiple mechanisms (i.e., AR amplification, mutations, alternative splicing and aberrant expression of AR co‐factors).^3^ Based on the theory of sustained AR signalling in CRPC, secondary generation AR pathway inhibitors (ARPIs) such as enzalutamide (Enza) or abiraterone have been put into clinical use. However, most patients still succumb when resistance to ARPIs happens.^4^ Therefore, identifying novel molecular mechanisms underlying the reinstated AR signalling is promising to improve the survival benefit of CRPC patients.

In addition to the genetic and epigenetic events occurring in the AR gene during PCa, post‐translational modifications (PTMs) of the AR protein can also profoundly alter its intrinsic biological activity.^5^ Several AR protein PTMs have been identified, including phosphorylation, methylation, acetylation, ubiquitylation and SUMOylation, among which phosphorylation is the most abundant.^5^ Most of the phosphorylated sites in AR protein are androgen activated, while some are androgen‐independently modulated.^5^, ^6^ For instance, serine 81 (S81) in the AR protein can be phosphorylated in response to androgen.^5^, ^6^ It has also been reported that several kinases from the cyclin‐dependent kinase (CDK) family, including CDK1, CDK2, CDK5 and CDK9, result in S81 phosphorylation.^5^, ^6^ Furthermore, androgen‐independent AR S81 phosphorylation enhances AR protein stability, promotes AR nuclear translocation and contributes to AR transactivation, leading to the continued AR signalling in CRPC.^7^, ^8^, ^9^

O‐GlcNAcylation is another type of PTM catalysed by O‐linked N‐acetylglucosamine (O‐GlcNAc) transferase (OGT), resulting in the transfer of O‐GlcNAc to serine/threonine residues of target proteins.^10^, ^11^, ^12^ Similar to phosphorylation, O‐GlcNAcylation routinely participates in various cellular processes by directly modifying transcription factors (TFs).^10^, ^11^, ^12^ Recent studies demonstrate a mechanism through which O‐GlcNAcylation modulates the function of TFs by competing with phosphorylation at the same or nearby serine/threonine residues.^13^ As a result, O‐GlcNAcylation and phosphorylation can be reciprocally regulated to coordinate the biological functions of their target proteins, as exemplified by the transactivation of the YAP protein via phosphorylation‐to‐O‐GlcNAcylation transition at threonine 241 during liver tumourigenesis.^14^ Although the AR phosphorylation sites are well documented, the O‐GlcNAcylation of the AR protein and the effects on CRPC progression are yet to be well studied.

Long non‐coding RNAs (lncRNAs) are a class of RNA transcripts with the length of more than 200 nt and have little ability to encode proteins but execute diverse biological processes, including tumourigenesis and drug resistance.^15^, ^16^ A increasing body of evidence has uncovered that lncRNAs are extensively participated in the pathogenesis of PCa.^15^, ^16^ In addition, a recent study declared that a group of 341 lncRNAs can be transcriptionally regulated by the AR protein.^17^ Their aberrant expression after AR‐targeted therapies, such as treatment with ADT and ARPIs, is accompanied by the progression of CRPC, indicating their critical role in this disease process.^17^, ^18^, ^19^ Several studies have highlighted the importance of lncRNAs in sustained AR signalling by elevating levels of AR transcripts or facilitating AR protein to bind to androgen‐responsive elements (AREs) of AR‐targeted genes.^18^, ^19^ However, the impacts of lncRNAs on the AR protein PTMs and their functional consequences are still unexplored to a great extent.

We discovered that lncRNA LINC01126 was significantly upregulated in metastatic PCa than in primary tumours and could predict poor prognosis in PCa patients. We first demonstrated that LINC01126 functions as an AR‐regulated lncRNA that is transcriptionally repressed by the AR protein, and thereby its expression can be elevated after AR‐targeted therapies. LINC01126, in turn, confers drug resistance to AR‐targeted therapies on PCa cells in vitro and in vivo. Mechanistically, LINC01126 controls the alteration of PTMs within AR protein by facilitating the transition from O‐GlcNAcylation at threonine 80 (T80) to phosphorylation at S81, resulting in enhanced AR protein stability, nuclear translocation and transactivation. In addition, LINC01126 facilitates the interaction of CDK9 with AR but impedes OGT binding with AR. These findings provide a novel insight into the progression of AR signalling‐dependent CRPC tumours and nominate LINC01126 as a candidate target to overcome treatment resistance.

## 2 METHODS

### 2.1 Bioinformatic analysis

Three publicly available PCa datasets, including GSE8511, Michigan Center for Translational Pathology (MCTP) cohort (GSE35988) and Memorial Sloan Kettering Cancer Center (MSKCC) cohort (GSE21034), were obtained from Gene Expression Omnibus (https://www.ncbi.nlm.nih.gov/geo/). Each of these datasets contains localised and metastatic tumour samples. With the cutoff criteria of adjusted *p*‐value <.05 and log_2_ fold change |FC| >1.5 for GSE8511 and GSE35988 and log_2_ |FC| >.5 for GSE21034, the differentially expressed genes (DEGs) of metastatic compared with localised tumours were identified using R package ‘limma’. The heatmap plots and volcano plots were created with the R package ‘pheatmap’ and ‘ggplot2’, respectively. Subsequently, the Venn analysis was conducted using the R package ‘Venn diagram’ to obtain upregulated genes distinguishing metastatic from localised tumours in all three datasets (upDEGs).

The RNA‐Seq and clinical data from PCa patients in the Cancer Genome Atlas (TCGA) were collected from the UCSC Xena online platform.^20^ The prognostic value of the upDEGs in the MSKCC and TCGA cohort was investigated using univariate Cox regression analysis. A Kaplan–Meier plot of survival curves was used to estimate the probability of biochemical recurrence (BCR), recurrence‐free survival and overall survival of the patients with low‐LINC01126 and high‐LINC01126 expression in the MSKCC and TCGA cohorts. Then, gene set enrichment analysis (GSEA) was conducted using the R package ‘cluster Profiler’. All R codes used in the research are available from the corresponding author upon reasonable request.

The binding propensity of the CDK family members and LINC01126 and the possible binding regions were estimated using algorithms provided by catRAPID (http://service.tartaglialab.com/page/catrapid_group).

### 2.2 Human samples

Tumour specimens with complete clinicopathological data were got from the Department of Urology, Xiangya Hospital, Central South University (CSU). Hormone‐sensitive prostate cancer (HSPC) samples were obtained by radical prostatectomy, while CRPC specimens were obtained by transurethral resection of the prostate to relieve bladder outlet obstruction. These samples were formalin‐fixed, paraffin‐embedded (FFPE). When necessary, the samples were further processed for haematoxylin and eosin (H&E) staining and RNA in situ hybridisation (ISH) assays. This study was approved by the Research Ethics Committee of Xiangya Hospital of CSU (2021101079) and informed consent was obtained from each patient.

### 2.3 Cell lines, cell culture and reagents

Human PCa (LNCaP, VCaP, C4‐2B, 22Rv1, DU145 and PC‐3) and human embryonic kidney 293T cell lines were obtained from the American Type Culture Collection. The cells were cultured under standard conditions as previously described.^21^ Cells were grown in phenol red‐free Roswell Park Memorial Institute (RPMI)‐1640 medium (Corning) supplemented with 10% charcoal‐stripped serum (Hyclone), mimicking an androgen‐deprived environment for experimental purposes. Cells were treated with Thiamet G (MedChemExpress) at a final concentration of 25 µM, OSMI‐1 (MedChemExpress) at a final concentration of 20 µM, MDV3100 (MedChemExpress) at a final concentration of 10 µM, dihydrotestosterone (DHT, Sigma–Aldrich) at a final concentration of 10 nM, actinomycin D (MedChemExpress) at a final concentration of 5 µg/mL, cycloheximide (CHX) at a final concentration of 10 µg/mL, BAY1251152 (Selleckchem) at a final concentration of 10 µM and uridine diphospho (UDP)‐GlcNAc (Sigma) at a final concentration of 60 mM. All cell lines were authenticated with short tandem repeat profiling and accepted regular tests for *mycoplasma* contamination.

### 2.4 RNAi, plasmid construction and cell transfection

Antisense oligos (ASO) targeting LINC01126 for sterically blocking its interaction with the CDK9 protein, ASO with the target sequence mutated, and ASO with non‐target sequence were designed with 2′‐O‐methoxyethyl modification and synthesised by GenePharma (Shanghai), while Dharmacon provided small‐interfering RNA (siRNA) pools targeting OGT, AR or CDK9. The lentiviral‐based LINC01126 expression plasmid and vectors used for short hairpin RNA (shRNA) targeting LINC01126 were obtained from Origene (Beijing). The best plasmid for efficient gene knockdown was selected for lentivirus packaging. Flag‐tag CDK9 expression plasmids were obtained from Addgene. The wild‐type (WT), S81A, S81D, S81A/T80A and S81A/S213A‐AR/AR‐V7‐GFP expression plasmids were constructed using the pEGFP‐C1 plasmid as the backbone. The WT, T80A, S81A, S115A, S201A and S213A‐AR‐HA expression plasmids were constructed using the pCMV‐N‐HA plasmid as the backbone. The His‐tagged OGT expression plasmid was constructed using the pCMV‐N‐His plasmid as the backbone. The KLK3‐luc reporters (WT or mutant) were constructed based on the pGL3‐Basic plasmid. The mutant reporter was constructed by inserting KLK3 promoter sequences without AREs. All these plasmids were constructed by GenePharma. Transfections with ASO were conducted with Lipofectamine RNAiMAX following the manufacturer's instructions. The methods for transient siRNA and DNA transfection were introduced previously.^21^ The methods for lentivirus packaging and establishment of stable cell lines were also previously described.^21^ Information on ASO, siRNAs and expression plasmids is described in Supporting Information S1.

### 2.5 Animal studies

The animal procedures were performed following the guidelines of the National Institutes of Health Guide for the Care and Use of Laboratory Animals and approved by the CSU Animal Ethics Committee (2020sydw0041). Briefly, 1 × 10^6^ PCa cells were resuspended in 100 µL of phosphate‐buffered saline (PBS) with 50% Matrigel (Corning) and randomly implanted subcutaneously into bilateral flank regions of 6‐week‐old male athymic nude mice nonobese diabetic/severe combined immunodeficient (NOD/SCID) with/without surgical castration according to the study design. A blinded researcher monitored tumour volume every third day using calipers and calculated using the equation: 1/2 × length × width^2^. At the endpoint, tumour samples were harvested, paraffin fixed and processed for immunohistochemical (IHC) analysis.

### 2.6 Subcellular fractionation

Nuclear and cytoplasmic protein extraction was performed using a NucBuster Kit (MerckMillipore) according to the manufacturer's instructions, and then western blotting was performed.

### 2.7 Detection of gene and protein expression by quantitative real‐time polymerase chain reaction and western blotting

The quantitative real‐time polymerase chain reaction (qRT‐PCR) and western blotting were performed with methods previously described.^21^ Information on all primary and secondary antibodies and their dilutions is listed in the Supporting Information S2. The primer sequences for each gene are also listed in the Supporting Information S3.

### 2.8 Cell viability, invasion and migration assays

The cell counting kit‐8 (CCK‐8) cell viability assay was introduced previously.^22^ In brief, cells in 96‐well plates were blended with CCK‐8 solution (Beyotime) for incubation, and absorbance was tested at 450 nm wavelength. The cell invasion assay has been previously described in detail.^22^ Starved cells were resuspended by using serum‐free culture medium and seeded into the Matrigel (Corning) coated upper transwell insert. Meanwhile, culture medium with 10% foetal bovine serum was used as a chemoattractant in the lower chamber. After incubation, the invading cells attached to the lower membrane of the insert were fixed, stained, imaged and counted. The wound‐healing assay was employed to measure the migration ability of cells. In brief, cells were seeded in six‐well plates and cultured to 90% confluence. An artificial scratch was made with a 10 µL pipette tip, and the cells were cultured in a serum‐free medium. Wound closure was observed and imaged in the same field under magnification after 48 h.

### 2.9 IHC staining and LINC01126 ISH

FFPE sections with human or xenograft tissues were deparaffinised, hydrated and stained with H&E. Full details on IHC staining have been given in previous studies.^21^ All detailed information on antibodies can be found in the Supporting Information S2. The ISH assay was performed with a DIG‐labelled LINC01126 probe (Supporting Information S1) using a customised kit from Boster. Based on instructions of the manufacturer, the deparaffinised sections were digested with proteinase K and hybridised with the probe at 52°C overnight. The slides were then visualised with an anti‐DIG‐POD antibody and DAB complex. Protein and LINC01126 expression levels were quantified based on their positive percentage and colour intensity.

### 2.10 RNA fluorescent ISH

The fluorescent ISH (FISH) kit was purchased from Ribo Bio, and experiments were performed following the manufacturer's instructions. Briefly, PCa cells were fixed with paraformaldehyde, treated with .5% Triton X‐100 in PBS and incubated in a pre‐hybridisation buffer. Finally, the cells were hybridised with CY3‐labelled U6 and LINC01126 FISH probes (Ribo Bio). Then, the cells were examined with a Zeiss confocal microscope (Oberkochen). The sequences of LINC01126 and U6 probes are listed in the Supporting Information S1.

### 2.11 Immunofluorescence

The immunofluorescence (IF) assay has been previously described.^22^ Briefly, cells were fixed in 4% paraformaldehyde, permeabilised with .5% Triton X‐100, blocked with 3% bovine serum albumin in phosphate buffered solution with Tween‐20 (PBST) and incubated with the primary antibody in the blocking buffer. Slides were washed with PBST, incubated in Alexa Fluor‐labelled secondary antibody in blocking buffer and counterstained with 4',6‐diamidino‐2‐phenylindole (DAPI) (ThermoFisher). Then, the cells were examined by a Zeiss LSM 510 confocal microscope. Antibodies of IF are listed in the Supporting Information S2.

### 2.12 Chromatin immunoprecipitation assay

Based on our established protocol, the chromatin immunoprecipitation (ChIP) assays were performed with the EZ ChIP Assay Kit (MerckMillipore).^22^ Briefly, cultured cells were crosslinked with formaldehyde, lysed with lysis buffer and sonicated using a microtip sonicator (Model 120, Fisher). Immunoprecipitation was performed using a ChIP‐grade anti‐AR antibody and immunoglobulin G (IgG). Precipitated DNA was quantified using qRT‐PCR and normalised to the respective input. ChIP‐qPCR primers targeting the promoter proximal region of genes are listed in the Supporting Information S3.

### 2.13 RNA immunoprecipitation assay

As introduced previously, the RNA immunoprecipitation (RIP) assays were conducted using the EZ‐Magna RIP RNA‐Binding Protein Immunoprecipitation Kit (MerckMillipore) following instructions of the manufacturer.^22^ In brief, cells were cultured, collected and lysed with RIP lysis buffer. The cell extract was incubated with magnetic beads conjugated to anti‐CDK9 or anti‐IgG antibody at 4°C overnight. Subsequently, the protein beads were eluted, precipitated RNA was purified, and qRT‐PCR was employed to detect LINC01126.

### 2.14 RNA pull‐down and mass spectrometry

In vitro transcription products of LINC01126 with 3′‐end biotinylation labelling were generated and provided by Genepharma. RNA pull‐down was conducted with the Pierce Magnetic RNA‐protein Pull‐Down Kit (ThermoFisher) following the manufacturer's instructions. In brief, each biotin‐labelled LINC01126 probe was incubated with cell protein extract. RNA/protein complexes were then isolated using streptavidin‐conjugated magnetic beads. After that, the complexes were washed and eluted by denaturation in the protein loading buffer. Samples were detected by western blot or silver staining. The differential band between sense and antisense LINC01126, indicated by silver staining, was clipped and processed for mass spectrometry (MS) analysis and retrieved from the human proteomics library.

### 2.15 Luciferase reporter assay

Control and LINC01126‐overexpression LNCaP or C4‐2B cells were transfected with KLK3‐luc reporter (WT or mutant) along with pRL‐TK and treated with or without MDV3100 to determine the effect of LINC01126 on KLK3‐luc reporter. As we introduced previously, the cells were harvested, and cell lysates were tested for relative luciferase activity using a Dual‐Luciferase Reporter Assay (Promega), as we previously described.^22^

### 2.16 Co‐immunoprecipitation assay

Based on the instruction from manufacturer, the co‐immunoprecipitation (Co‐IP) assays were strictly carried out by using the Protein A+G Agarose Gel Immunoprecipitation Kit (Beyotime). Cells were incubated with protein A+G Agarose Gel and lysis buffer with or without .1% SDS (used for denatured IP [for O‐GlcNAc] or conventional IP, respectively). The immunoprecipitates were washed and then subjected to western blotting. The antibodies are listed in the Supporting Information S2.

### 2.17 Statistics

All data shown in the figures represent three or more independent experimental times. Data are expressed as the mean ± standard deviation or the mean ± standard error of the mean unless otherwise indicated. GraphPad Prism 5.01 software (GraphPad Software) was employed to perform statistical analyses. Two‐tailed Student's *t*‐test was performed to analyse the differences in the mean between two groups. Survival curves were plotted by using Kaplan–Meier, while the relationship between two expression samples was defined using spearman's *r* correlation coefficient. *p*‐Value < .05 considered significantly different.

## 3 RESULTS

### 3.1 LINC01126 is elevated in CRPC, and AR directly regulates its transcription

**FIGURE 1:**
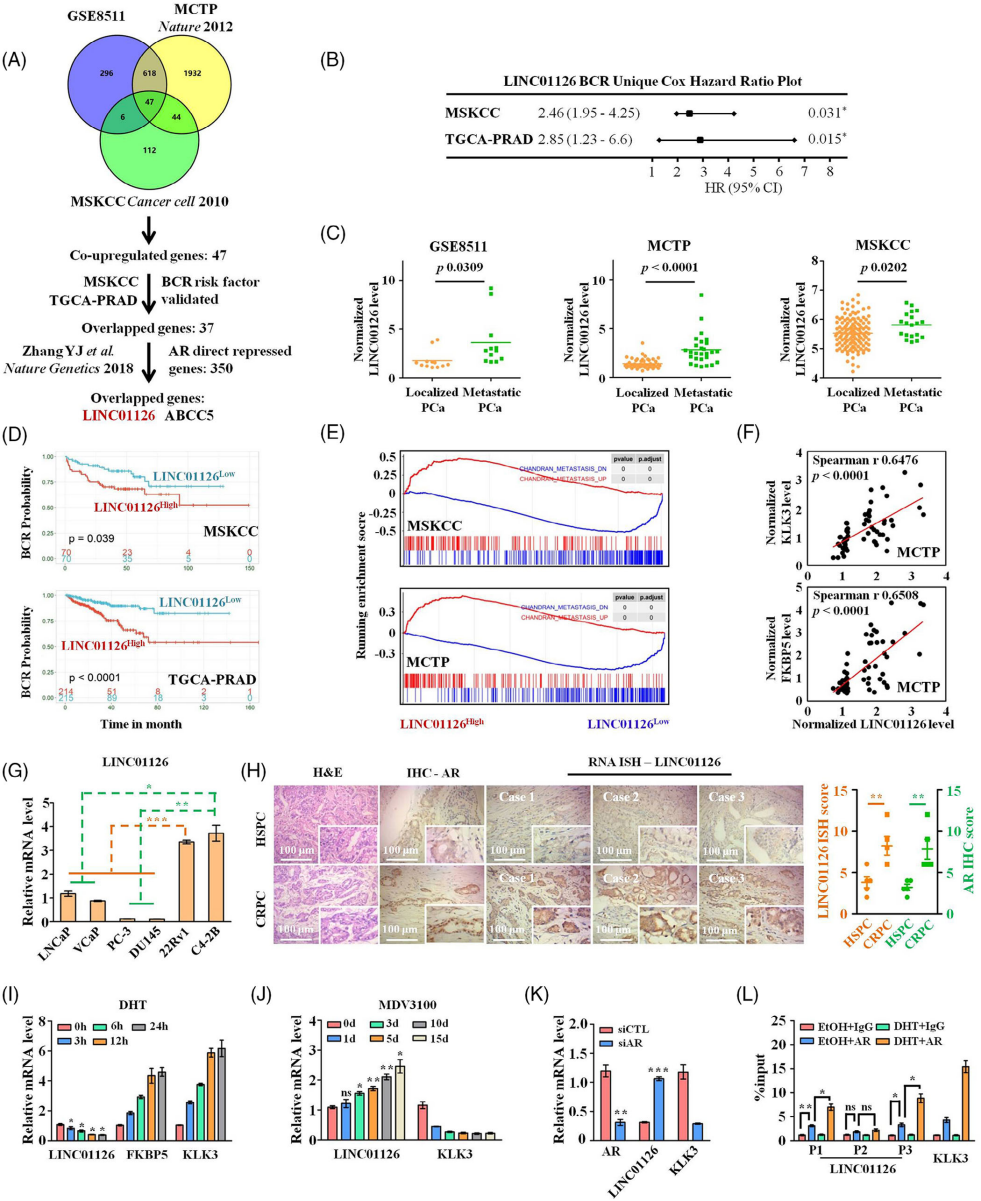
Androgen receptor (AR)‐related long non‐coding RNA (lncRNA) LINC01126 is elevated in castration‐resistant prostate cancers (CRPC). (A) A schematic illustration of the screening process for target lncRNA. (B) LINC01126 was confirmed to be a risk factor of biochemical recurrence (BCR) by univariate Cox regression analysis in MSKCC and The Cancer Genome Atlas‐prostate adenocarcinoma (TCGA‐PRAD) cohorts. (C) A plot showing the LINC01126 expression in tumour tissues derived from GSE8511, Michigan Center for Translational Pathology (MCTP) and Memorial Sloan Kettering Cancer Center (MSKCC) clinical prostate cancer (PCa) cohorts. (D) Kaplan–Meier curves for the high‐LINC01126 and low‐LINC01126 groups. Patients in the high‐LINC01126 group had a higher risk for BCR compared with those in the low‐LINC01126 group (*p* = .039 for MSKCC cohort and *p* < .0001 for TCA‐PRAD cohort). (E) Gene set enrichment analysis (GSEA) revealed the enrichment of the ‘CHANDRAN_METASTASIS_UP’ gene signature in the transcriptome of PCa tumours in the high‐LINC01126 group relative to the low‐LINC01126 group. (F) Spearman correlation analysis confirmed that LINC01126 expression in tumour tissues derived from MCTP clinical PCa cohort was positively correlated with the messenger RNA (mRNA) expression of KLK3 and FKBP5. (G) The expression level of LINC01126 was measured by quantitative polymerase chain reaction (qPCR) in a panel of PCa cell lines as indicated. Data between two groups were analysed with unpaired Student's *t*‐test. (H) RNA in situ hybridisation (ISH) detection of LINC01126 expression and immunohistochemistry (IHC) detection of AR protein in hormone‐sensitive prostate cancer (HSPC) and AR‐dependent CRPC tumours. Scale bar: 100 µm. Left panel: representative images; right panel: statistical analysis of specimens from five HSPC and five CRPC patients, respectively. (I and J) Effects of AR ligand dihydrotestosterone (DHT, 10 nM) and AR antagonist MDV3100 (10 µM) on LINC01126 expression in LNCaP cells were determined by qPCR. Data between 0 h or 0 d group and another group were analysed with unpaired Student's *t*‐test. (K) LINC01126 expression level in LNCaP cells with/without AR knockdown was quantified through qPCR. (L) Chromatin immunoprecipitation (ChIP)‐qPCR showing the enrichment of AR at two distinct genomic sites (P1 and P3) located within the LINC01126 promoter upon DHT (10 nM) stimulation in LNCaP cells. The KLK3 promoter proximal region was employed as a positive control for DHT treatment. CTL, control; H&E, haematoxylin and eosin. ns, not significant, ^*^ *p* < .05, ^**^ *p* < .01 and ^***^ *p* < .001.

We analysed three previously published RNA‐Seq data from clinical PCa cohorts that included localised and metastatic tumours (GSE8511, MCTP cohort and MSKCC cohort) to screen the possible genes that may contribute to the progression of PCa (Figure S1A,B). The results showed that 47 genes were consistently upregulated in metastatic tumours in these cohorts. The univariate Cox regression analysis demonstrated that 37 of the 47 genes increased the risk of BCR in MSKCC and TCA‐PRAD cohorts (Table S1 and Figures 1A,B and S1C). Zhang et al. recently reported that the AR directly repressed 350 genes (Table S2).^17^ Theoretically, these genes can be induced by AR‐targeted therapies and play critical roles in the development of CRPC. Of the 37 genes, two (LINC01126 and ABCC5) caught our interest as they were also reported by Zhang et al. to be AR‐direct repressed genes (Figure 1A). While the function of ABCC5 in promoting CRPC progression has been well studied,^7^ the clinical relevance of LINC01126 in PCa progression remains largely unknown.

LINC01126 (ENSG00000279873) is located on chromosome (chr) 2p21 and has an annotated transcript in the Gene code database (GENECODE V39, Figure S1D). The coding capability of LNC01126 was examined using the Coding Potential Assessment Tool and the Coding Potential Calculator 2. The results showed that LINC01126 has no protein‐coding possibility (Figure S1E). Recent studies explained that LINC01126 plays a role in predicting the survival benefit of patients with advanced melanoma^23^ and may promote the pathogenesis of periodontitis.^24^ The bioinformatics analysis indicated that LINC01126 might be critical in PCa progression. We found that LINC01126 was preferentially expressed in metastatic CRPC tumours (Figure 1C). Moreover, higher LINC01126 expression in tumours was associated with a significantly worse prognosis, including shorter BCR, progression‐free and overall survival times visualised on Kaplan–Meier curves (Figures 1D and S2A). The GSEA also showed that genes correlated with metastases are more highly enriched in the transcriptome, mediated by high LNC01126 expression in PCa (Figure 1E and Tables S3 and S4). In addition, LINC01126 was positively correlated with two canonical markers of AR signalling, including KLK3 and FKBP5 (Figures 1F and S2B). The bioinformatics analysis results were validated by performing RNA FISH, which verified the expression of LINC01126 in PCa cells. Interestingly, LINC01126 was mostly observed in the cytoplasm of the hormone‐sensitive LNCaP cells but had more pronounced expression in the nucleus of androgen‐independent CRPC cells, including 22RV1 and C4‐2B (Figure S2C). We further confirmed the overexpression of LINC01126 in CRPC cell lines and AR‐dependent CRPC tumours with qRT‐PCR and RNA ISH assays (Figure 1G,H).

LINC01126 was identified as a potential direct AR‐repressed lncRNA using RNA‐Seq and ChIP‐Seq data.^17^ We checked LINC01126 expression in hormone‐sensitive LNCaP cells exposed to androgen or AR antagonists to demonstrate androgen regulation of LINC01126. As expected, adding DHT to LNCaP cells after 48 h of cultivation in an androgen‐deprived medium reduced the expression of LINC01126 in a time‐ and concentration‐dependent manner (Figures 1I and S2D). In contrast, MDV3100 (a potent AR antagonist) treatment induced chronic LINC01126 expression (Figure 1J). In addition, the knockdown of AR in LNCaP cells also elevated LINC01126 expression (Figure 1K). We analysed the AR ChIP‐Seq data from the Cistrome DB database to examine whether AR transcriptionally regulates LINC01126.^25^ The results showed that AR was primarily recruited at three different genomic sites within the LINC01126 promoter, including: (i) chr2:43226266‐43226304 (P1), (ii) chr2:43226859‐43226919 (P2) and (iii) chr2:43227080‐43227130 (P3). ChIP‐qPCR assays for AR in DHT‐stimulated LNCaP cells were then employed to confirm the occupancy of AR at the three sites, and significant enrichment in AR binding at P1 and P3 was observed (Figure 1L). These findings demonstrate that LINC01126 acts as a direct AR‐regulated lncRNA induced by AR‐targeted therapies and upregulated in the CRPC stage of PCa, suggesting a functional significance of LINC01126 gain in CRPC formation.

### 3.2 LINC01126 contributes to the development of androgen‐independence in PCa cells

**FIGURE 2:**
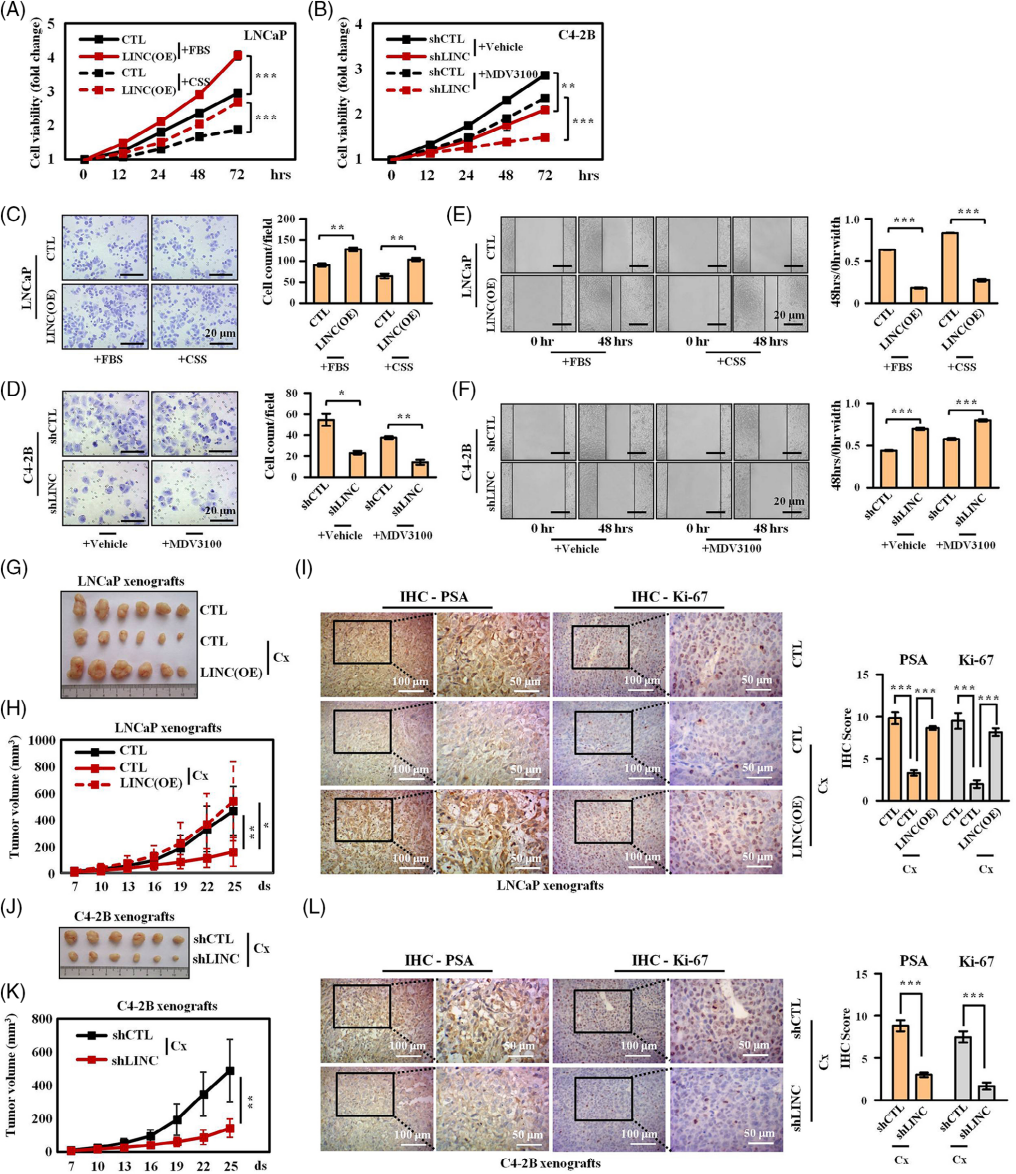
LINC01126 is required for androgen‐independent trait and aggressive behaviours of prostate cancer (PCa) cells. (A) cell counting kit‐8 (CCK‐8) assays for the viability of cells in the control and LINC01126‐stable overexpression LNCaP cells in full and androgen‐deprived medium. (B) CCK‐8 assays for cell viability in the control and LINC01126‐stable knockdown C4‐2B cells without and with MDV3100 treatment (10 µM). Data are presented as the fold changes in cell viability during an observation period of up to 72 h, and are representative of three replicate experiments (*n* = 3). Fold change on the day of cell seeding (hour 0) in each group was set as 1. (C) Representative images (left) and quantification of the invaded cells (right) derived from transwell assays of control and LINC01126‐overexpression LNCaP cells in culture medium as indicated (*n* = 3). Scale bar: 20 µm. (D) Representative images (left) and quantification of the invaded cells (right) derived from transwell assays of control and LINC01126‐knockdown C4‐2B cells without and with MDV3100 treatment (10 µM) for 48 h (*n* = 3). Scale bar: 20 µm. (E) Representative images (left) and quantification of cell migration (right) derived from wound healing assays of control and LINC01126‐overexpression LNCaP cells in culture medium as indicated (*n* = 3). Scale bar: 20 µm. (F) Representative images (left) and quantification of cell migration (right) derived from wound healing assays of control and LINC01126‐knockdwon C4‐2B cells in the same context as D (*n* = 3). Scale bar: 20 µm. Representative images (G) and growth curve (H) of tumour xenografts in male nude mice with or without castrated surgery 25 days after subcutaneous inoculation with control and LINC01126‐overexpression LNCaP cells. (I) The expression level of PSA and Ki‐67 in the xenografts in G was determined by immunohistochemistry (IHC) staining, and the IHC scores were quantified. Representative images (J) and growth curve (K) of tumour xenografts in castrated male nude mice 25 days after subcutaneous inoculation with control and LINC01126‐knockdown C4‐2B cells. (L) PSA and Ki‐67 from the xenografts in (J) were measured by IHC staining and scored. Scale bar: 50/100 µm as indicated. CSS, charcoal‐stripped serum; CTL, control; Cx, castration; FBS, foetal bovine serum; LINC(OE), LINC01126 overexpression; shCTL, shControl; shLINC, shLINC01126. ^*^ *p* < .05, ^**^ *p* < .01 and ^***^ *p* < .001.

Given the positive relationship between the LINC01126 expression level and PCa progression, we further evaluated the biological effects of LINC01126 on PCa cells. As shown in Figure 1G, the hormone‐sensitive LNCaP cells express a moderate level of LINC01126, whereas androgen‐independent C4‐2B cells express the highest level of LINC01126 compared to other PCa cell lines. Therefore, we established stable LINC01126 overexpressing and knockdown cells in LNCaP and C4‐2B cells, respectively, and validated the LINC01126 expression levels by qRT‐PCR (Figure S3A,B). Our results from CCK‐8, invasion and migration assays showed that stable LINC01126 overexpression significantly enhanced the cell proliferation, invasion and migration of LNCaP cells, both in complete medium and androgen‐depleted medium (Figure 2A,C,E). In contrast, the knockdown of LINC01126 significantly repressed the cell proliferation, invasion and migration of C4‐2B cells, in both the complete medium and the presence/absence of the potent AR antagonist, MDV3100 (Figure 2B,D,F). As expected, LINC01126 silencing also compromised the resistance of CRPC 22Rv1 cells to Enza (MDV3100, Figure S3C–F).

In the subsequent in vivo study, subcutaneous xenografts were established in immunodeficient mice, with/without surgical castration, using cells with stable overexpression of LINC01126 or shRNA targeting LINC01126. We observed that elevated LINC01126 expression significantly promoted the growth of LNCaP tumours in castrated mice (Figure 2G,H). To further investigate the underlying mechanism, we evaluated the expression of the proliferation marker Ki‐67 and the canonical AR‐activated gene PSA/KLK3 by IHC staining in LNCaP tumours (Figure 2I). The results further supported that LINC01126 contributed to tumour growth by reactivating AR signalling after castration, which is widely accepted as a primary mechanism for CRPC formation. Conversely, the knockdown of LINC01126 impeded the growth of C4‐2B tumours in castrated conditions by repressing AR signalling (Figure 2J–L). These data, collected in both in vitro cell lines and in vivo mice models, consistently support the idea that LINC01126 contributes to the development of androgen independence in PCa cells, indicating its significance in CRPC progression.

### 3.3 LINC01126 activates the AR protein through a post‐translational mechanism

**FIGURE 3:**
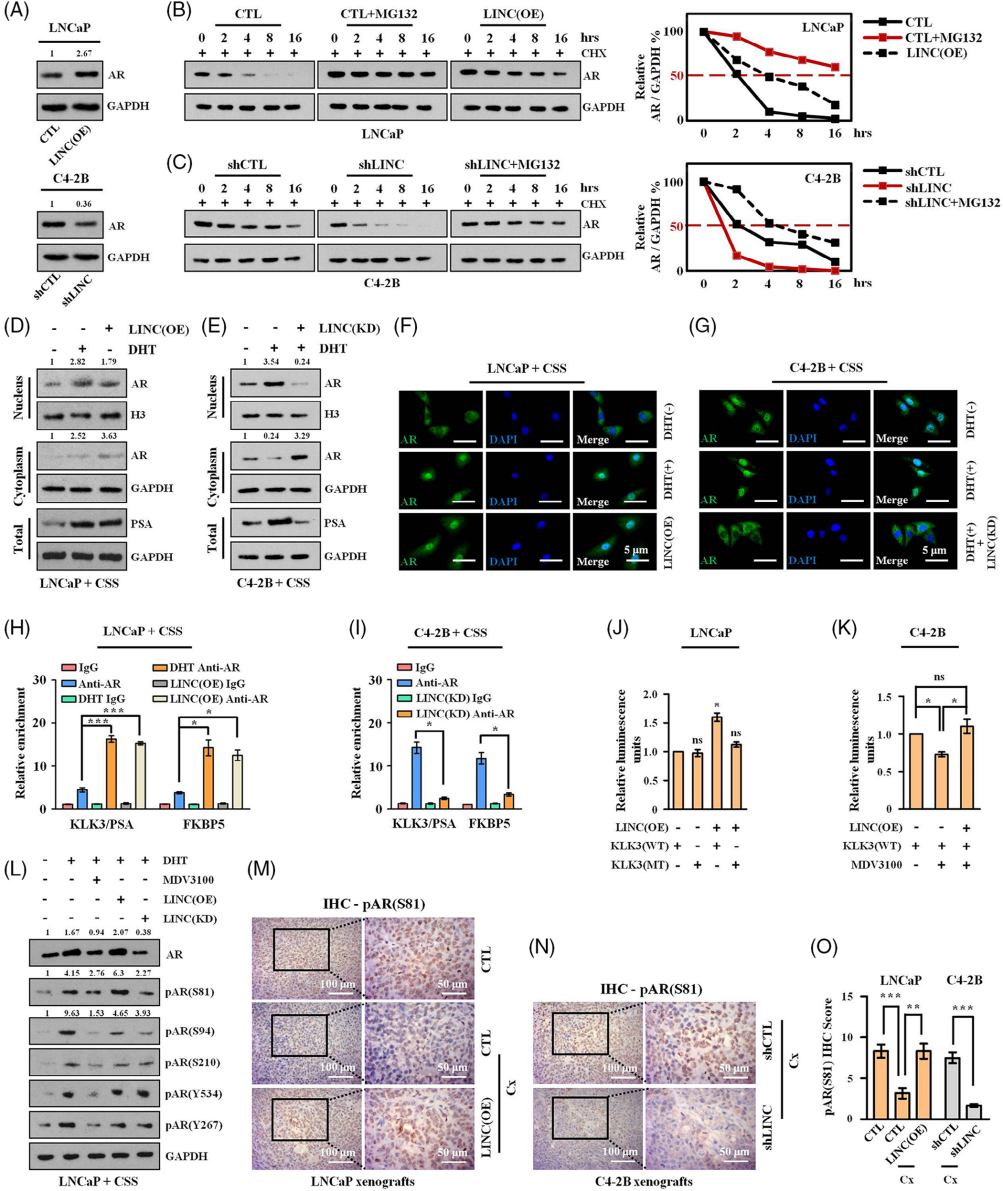
LINC01126 facilitates activation of the androgen receptor (AR) protein. (A) Immunoblotting results for AR protein level in LNCaP cells with or without (w/o) LINC01126 overexpression and in C4‐2B cells w/o LINC01126 knockdown. (B and C) Protein synthesis was blocked by treatment with cycloheximide (CHX) (10 µg/mL) for the indicated times to test AR stability. The ubiquitin–proteasome system inhibitor MG132 (10 µM) was added as indicated. Endogenous AR protein level in LNCaP cells w/o LINC01126 overexpression (B) and in C4‐2B cells w/o LINC01126 knockdown (C) was measured and quantified by immunoblotting. (D–I) LNCaP cells w/o LINC01126 overexpression (D, F and H) and C4‐2B cells w/o LINC01126 knockdown (E, G and I) were cultured in androgen‐deprived medium for 24 h. The cells were subsequently cultured w/o dihydrotestosterone (DHT) (10 nM) treatment for 24 h. (D and E) Next, nuclear and cytoplasmic protein extraction were performed. AR protein level in cytoplasm and nucleus was determined by immunoblotting. PSA protein level in whole cell lysis was also measured. (F and G) Confocal microscopic images showing the localisation of AR. Scale bar: 5 µm. (H and I) Chromatin immunoprecipitation (ChIP)‐quantitative polymerase chain reaction (qPCR) data illustrating the enrichment of AR on the promoter proximal regions of the KLK3 and FKBP5 genes. (J) The luciferase signal in LNCaP cells w/o LINC01126 transient overexpression was determined by luciferase reporter assays employing wild‐type or mutant KLK3 reporter vectors. Data between the first group and another group were analysed with unpaired Student's *t*‐test. (K) C4‐2B cells w/o LINC01126 transient overexpression were treated with MDV3100 (10 µM) for 24 h. The luciferase signal in cells was determined by luciferase reporter assays employing wild‐type KLK3 reporter vectors. (L) LNCaP cells w/o LINC01126 transient overexpression and knockdown were cultured in androgen‐deprived medium for 24 h. This was followed by culture w/o DHT (10 nM) and MDV3100 (10 µM) treatment for 24 h. AR protein level and its phosphorylation level at several sites (serine 81 [S81], S94, S210, Y534 and Y267) were then measured by immunoblotting. (M and N) AR S81 phosphorylation was measured and quantified (O) from the xenografts described in Figure 2G,J by immunohistochemistry (IHC) staining. Scale bar: 50/100 µm as indicated. CSS, charcoal‐stripped serum; CTL, control; Cx, castration; LINC(KD), LINC01126(KD); LINC(OE), LINC01126 overexpression; shCTL, shControl; shLINC, shLINC01126. ns, not significant, ^*^ *p* < .05, ^**^ *p* < .01 and ^***^ *p* < .001.

While exploring the biological functions of LINC01126, we fortuitously discovered that LINC01126 overexpression had almost no impact on cell proliferation in AR‐negative PCa cell lines (PC‐3 and DU145, Figure S4A,B). More intriguingly, we found that AR silencing by a pool of siRNA targeting AR messenger RNA (mRNA) prevented the proliferation of the AR‐positive LNCaP cells overexpressing LINC01126 (Figure S4C). Combined with the finding that LINC01126 activated the AR‐mediated gene KLK3/PSA in mice, we postulated that the function of LINC01126 might be mediated, at least in part, by AR. While transcriptional regulation is one of the most important mechanisms of action of lncRNAs,^16^ we were unable to detect any changes in the level of AR mRNA, and its mRNA stability remained unaltered when LINC01126 was overexpressed in LNCaP cells and suppressed in C4‐2B cells, ruling out the possibility that LINC01126 regulated the mRNA level of AR (Figure S4D,E). We then evaluated the protein level of AR in the same setting and found that LINC01126 positively regulated AR protein level (Figure 3A). Additionally, we confirmed through CHX chase experiments that LINC01126 overexpression and the ubiquitin–proteasome system (UPS) inhibitor MG132 had a comparable impact on protecting AR protein from degradation in LNCaP cells (Figure 3B). Moreover, knockdown of LINC01126 in C4‐2B cells decreased the half‐life of AR protein, which could be reversed by UPS inhibitor MG132 (Figure 3C). We also observed similar results when exogenous AR with a green fluorescent protein (GFP)‐tag was transfected into LNCaP and C4‐2B cells in the same setting, further verifying the finding that LINC01126 prolonged the half‐life of AR protein (Figure S4F).

AR, a ligand‐regulated TF belonging to the nuclear receptor superfamily,^5^ comprises three dominant structures that are the N‐terminal domain (NTD), the DNA‐binding domain and the ligand‐binding domain.^5^ When not bound to a ligand (androgen), AR is inactive and primarily localised in the cytoplasm. However, upon ligand binding, AR translocates into the nucleus where it binds DNA to exert its function as a TF.^5^ In our study, we subsequently investigated whether LINC01126 affects AR activation. We employed cytoplasmic/nuclear separation analysis and IF assay to observe the nuclear translocation of AR mediated by androgen or LINC01126. We found that LINC01126 overexpression mimicked the effect of androgen on AR transportation in LNCaP cells (Figure 3D,F). Moreover, we observed that decreased LINC01126 expression correlated with a decreased AR nuclear translocation mediated by androgen in C4‐2B cells (Figure 3E,G). KLK3/PSA and FKBP5 are two well‐known AR‐responsive genes that are transcriptionally activated by nuclear AR.^26^ We used ChIP‐qPCR assays for AR to confirm the occupancy of AR on the promoter proximal regions of the KLK3 and FKBP5 genes. We observed a marked enrichment for AR binding at the KLK3 and FKBP5 promoters when LINC01126 was elevated or androgen was supplied in androgen‐starved LNCaP cells (Figure 3H). Furthermore, we examined the occupancy of AR on the promoter region of the two genes in C4‐2B cells with LINC01126 knockdown and observed a profound decrease in recruitment, suggesting impaired AR binding (Figure 3I). To explore the impact of LINC01126 on the transcriptional level of the AR‐activated genes, we performed dual‐luciferase reporter assays in PCa cells, wherein WT/mutant KLK3 reporter plasmids were employed. We found that LINC01126 overexpression remarkably increased the luciferase signal from the WT KLK3 reporter plasmids, while the signal from the ARE‐mutant KLK3 reporter plasmids was not significantly changed by LINC01126 overexpression in LNCaP cells (Figures S4G and 3J). Moreover, MDV3100 attenuated the luciferase signal from the WT KLK3 reporter plasmids, but LINC01126 overexpression abrogated the impact of MDV3100 on the luciferase signal in C4‐2B cells (Figures S4H and 3K). As expected, qRT‐PCR assays confirmed that LINC01126 positively regulated the mRNA expression level of the KLK3 and FKBP5 genes (Figure S4I).

As reported, in addition to the transactivation by androgen, AR can be also regulated in an androgen‐independent manner by PTMs, of which phosphorylation accounts for the majority, and most phosphorylated sites are located in the NTD of AR.^5^, ^6^ These PTMs are essential for maintaining the protein stability, nuclear translocation and transcriptional activity of AR,^5^, ^6^ which are analogous to the functions of LINC01126 that we observed here. This prompted us to investigate whether LINC01126 activates AR through PTMs. We determined the most frequently phosphorylated sites located in the NTD of AR by immunoblotting and found that the level of S81and S94 phosphorylation might be affected by LINC01126 in LNCaP cells. However, when LINC01126 was either elevated or repressed, the most consistent and dramatic changes occurred in S81 site both in the presence and absence of DHT (Figures S4J,K and 3L). The positive regulation of S81 phosphorylation by LINC01126 was further verified in vivo in mice models through IHC staining (Figure 3M–O). Together, these findings indicate that LINC01126 stabilises the AR protein and activates AR by facilitating the phosphorylation of AR at S81.

### 3.4 LINC01126 facilitates CDK9 to enhance AR S81 phosphorylation

**FIGURE 4:**
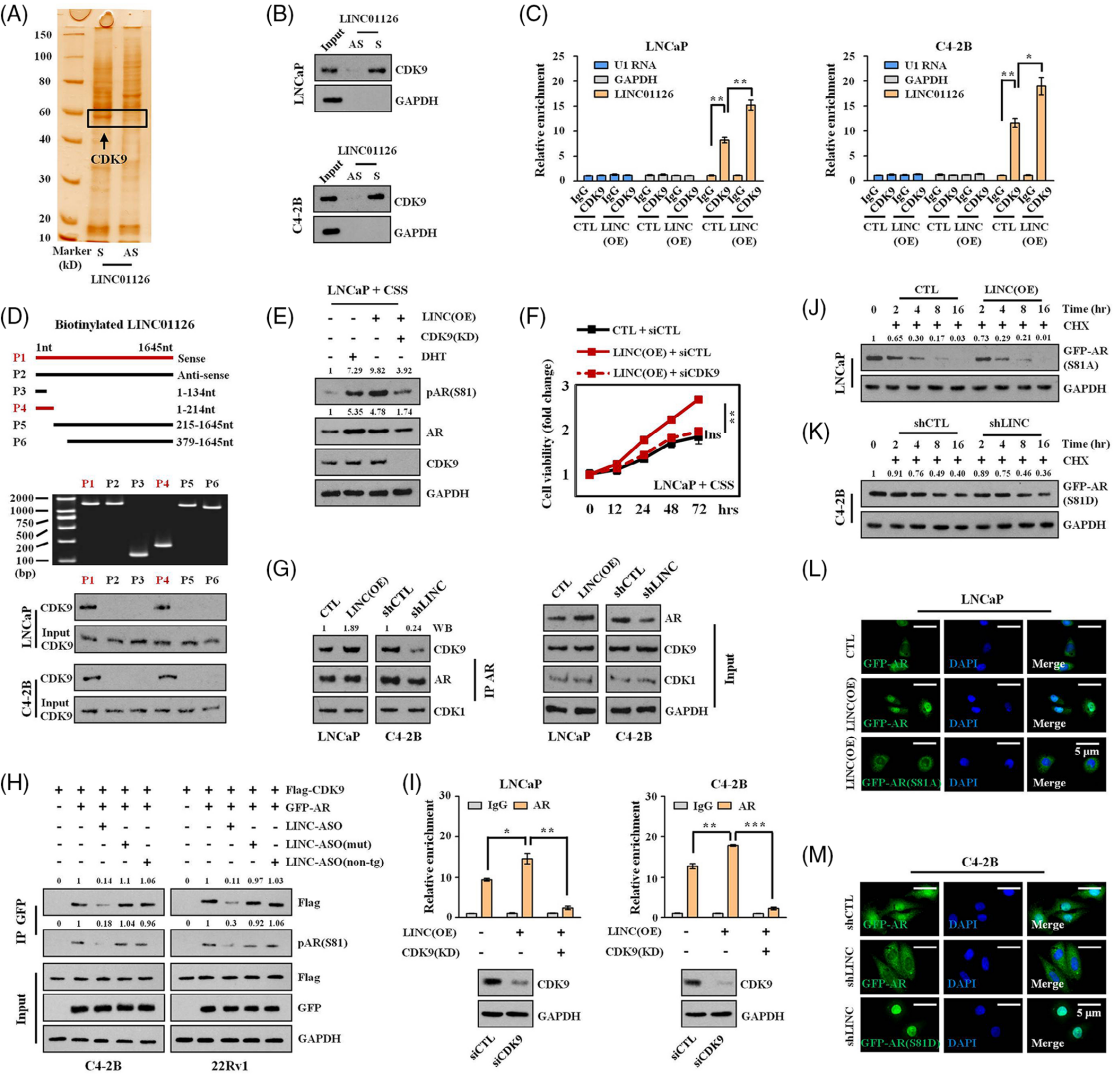
LINC01126 facilitates CDK9 to phosphorylate androgen receptor (AR) protein. (A) Identification of LINC01126‐associated proteins through RNA pull‐down assay. The proteins pulled down by LINC01126 or the antisense RNA of LINC01126 incubated with C4‐2B cell extracts were resolved by sodium dodecyl sulfate‐polyacrylamide gel electrophoresis (SDS‐PAGE) and subjected to silver staining. A specific band was identified in the LINC01126 group and is marked with a black box. (B) The interaction between LINC01126 and CDK9 was confirmed by RNA pull‐down followed by immunoblotting in LNCaP and C4‐2B cells. GAPDH was used as a negative control. (C) Real‐time quantitative polymerase chain reaction (qPCR) analysis of LINC01126 in RNA immunoprecipitation (RIP) assays of LINC01126 transient overexpression or control LNCaP and C4‐2B cells using anti‐CDK9, RNA enrichment was determined in reference to non‐immunised immunoglobulin G (IgG) control. U1 RNA and GAPDH were used as negative controls. (D) Up panel: schematic view of truncated LINC01126. Full length anti‐sense was used as the negative control. RNAs corresponding to indicated fragments were biotinylated and used in the RNA pull‐down assays to identify the binding domain of LINC01126 required for a physical interaction with CDK9. (E) LNCaP cells with or without (w/o) LINC01126 overexpression were cultured in androgen‐deprived medium for 24 h. The cells were then cultured w/o dihydrotestosterone (DHT) (10 nM) and small‐interfering RNA (siRNA) pool targeting CDK9 treatment for another 24 h. Immunoblotting assay was then performed to measure the AR and AR serine 81 (S81) phosphorylation level. (F) cell counting kit‐8CCK‐8) cell viability assay of control and LINC01126‐overexpression LNCaP cells that were cultured w/o siRNA pool targeting CDK9 treatment in androgen‐deprived medium. Data are presented as the fold changes in cell viability during an observation period of up to 72 h, and are representative of three replicate experiments (*n* = 3). Fold change on the day of cell seeding (hour 0) in each group was set as 1. (G) Binding ability of AR and indicated proteins using immunoprecipitation in LNCaP cells w/o LINC01126 overexpression and C4‐2B cells w/o LINC01126 knockdown. (H) C4‐2B and 22Rv1 cells were transfected with plasmid expressing AR‐GFP together with CDK9‐Flag plasmid. Then, cells were treated w/o antisense oligos (ASO) as indicated. GFP‐AR was immunoprecipitated with the anti‐green fluorescent protein (GFP) antibody and blotted for Flag and AR S81 phosphorylation to examine the interaction of exogenous AR and CDK9. (I) Real‐time qPCR analysis of LINC01126 in RIP assays of LINC01126 overexpression or control LNCaP (left panel) and C4‐2B (right panel) cells w/o siRNA pool targeting CDK9 using anti‐AR antibody, RNA enrichment was determined relative to the non‐immunised IgG control. (J and K) Protein synthesis was blocked by treatment with cycloheximide (CHX) (10 µg/mL) for the indicated times to test the stability of exogenous AR protein with amino acid mutation in LNCaP cells w/o LINC01126 overexpression (J) and in C4‐2B cells w/o LINC01126 knockdown (K). (L and M) Confocal microscopic images indicating the localisation of exogenous AR protein w/o amino acid mutation in LNCaP cells w/o LINC01126 overexpression (L) and in C4‐2B cells w/o LINC01126 knockdown (M). Scale bar: 5 µm. A, sense; AS, anti‐sense; CSS, charcoal‐stripped serum; CTL, control; LINC‐ASO (mut), LINC‐ASO (mutated); LINC‐ASO (non‐tg), LINC‐ASO (non‐targeted); LINC(OE), LINC01126 overexpression; shCTL, shControl; shLINC, shLINC01126. ns, not significant, ^*^ *p* < .05, ^**^ *p* < .01 and ^***^ *p* < .001.

As the most extensively studied phosphorylation site, AR S81 has been reported to be tightly associated with AR protein stability, nuclear retention and transactivation.^5^, ^6^ In addition to its response to androgen, S81 can also be phosphorylated by several kinases from the CDK family, including CDK1, CDK2, CDK5 and CDK9.^5^, ^6^ However, we observed that LINC01126 had no significant impact on the mRNA and protein levels of these kinases (Figure S5A,B). We then hypothesised that LINC01126 could contribute to S81 phosphorylation by facilitating the biological function of these CDK proteins. To delineate the mechanism underlying the effect of LINC01126 on AR phosphorylation, bioinformatic methods were first performed to evaluated the possible interaction between LINC01126 and these CDK proteins. Interestingly, the catRAPID program predicted all the CDK proteins mentioned above to interact with LINC01126 (Figure S5C,D). This motivated us to perform RNA pull‐down assays in vitro with biotinylated LINC01126, followed by sodium dodecyl sulfate‐polyacrylamide gel electrophoresis (SDS‐PAGE) electrophoresis. We detected an overtly differential band located between 40 and 60 kDa in the sense lane (Figure 4A). The gel was then subjected to MS to screen out the candidate proteins interacting with LINC01126. CDK9 and CDK7 (another member of the CDK family) were among the candidates obtained by MS, while CDK9 received the highest score in the list (Table S5). Next, we confirmed that CDK9 is the most robust LINC01126‐binding protein among the CDK proteins mentioned above using a biotin‐labelled RNA pull‐down followed by immunoblotting (Figures 4B and S5E). Furthermore, we verified the LINC01126–CDK9 interaction by a RIP‐qPCR assay. In comparison to IgG, CDK9 contributed to a significant enrichment of LINC01126 in both LNCaP and C4‐2 cells, especially under the condition of LINC01126 overexpression (Figure 4C). To determine the region of LINC01126 to which CDK9 binds, we prepared a series of biotin‐labelled LINC01126 probes with deletion mutants based on the prediction of the catRAPID program (Figure S5D) and performed in vitro RNA pull‐down experiments in LNCaP and C4‐2B cells. The results indicated that the 135−214 nucleotide position of LINC01126 was sufficient to bind CDK9 (Figure 4D).

Consistent with reports from other investigators,^8^ we confirmed that CDK9 enhances the phosphorylation of AR S81 in PCa cells (Figure S5F). We further demonstrated that CDK9 knockdown effectively reversed the AR S81 phosphorylation stimulated by LINC01126 (Figure 4E). Moreover, the results from the CCK‐8, invasion and migration assays showed that CDK9 knockdown abrogates the androgen‐independent features observed in LNCaP cells with stable LINC01126 overexpression (Figures 4F and S5G). These findings indicate that CDK9 functions as a downstream effector of LINC01126. This was further supported by Co‐IP experiments in LNCaP and C4‐2 cells, which consistently indicated that LINC01126 enhances the interaction of CDK9 with AR (Figure 4G). We also confirmed this phenomenon using exogenous proteins by transfection in the same setting to exclude the interference from changes of endogenous AR protein level (Figure S5H,I). Based on the nucleotide sequence in LINC01126 that was confirmed to bind CDK9, we designed and constructed an ASO specifically targeting the binding sequence (LINC‐ASO), an ASO with the target sequence mutated (LINC‐ASO [mutated]) and an ASO with non‐target sequence (LINC‐ASO [non‐targeted]). We confirmed that the LINC01126‐ASO could not change the level of LINC01126 in C4‐2 and 22Rv1 cells, excluding the possibility of LINC01126 degradation (Figure S5J). We found that the LINC‐ASO, not the mutated or non‐targeted ASO, had the ability to effectively attenuate the interaction of exogenous CDK9 with exogenous AR, and inhibit the phosphorylation of AR S81 (Figure 4H). This function of LINC‐ASO was further verified in the endogenous AR and CDK9 proteins (Figure S5K). On the other hand, in the RIP‐qPCR assays for detecting the LINC01126‐AR interaction, we confirmed that CDK9 knockdown also attenuates the interaction of LINC01126 with AR (Figure 4I).

As previously mentioned, the consequence of the CDK9 interaction with AR is the phosphorylation of AR at S81. We then replaced S81 with either alanine (S81A mutant) or aspartate (S81D mutant) to inactivate or maintain AR phosphorylation, respectively, whether CDK9 was interacting or not. We found that the enhanced stability of AR protein and AR nuclear translocation due to LINC01126 overexpression was abrogated when S81 phosphorylation was inactivated by mutation (Figure 4J,L). Additionally, the impairment of AR activation by LINC01126 knockdown was blocked when S81 phosphorylation was sustained (Figure 4K,M). These data together demonstrate that LINC01126 contributes to AR S81 phosphorylation by facilitating the interaction between CDK9 and AR.

### 3.5 O‐GlcNAc modification of AR at T80 exerts an inhibitory effect on AR activation

**FIGURE 5:**
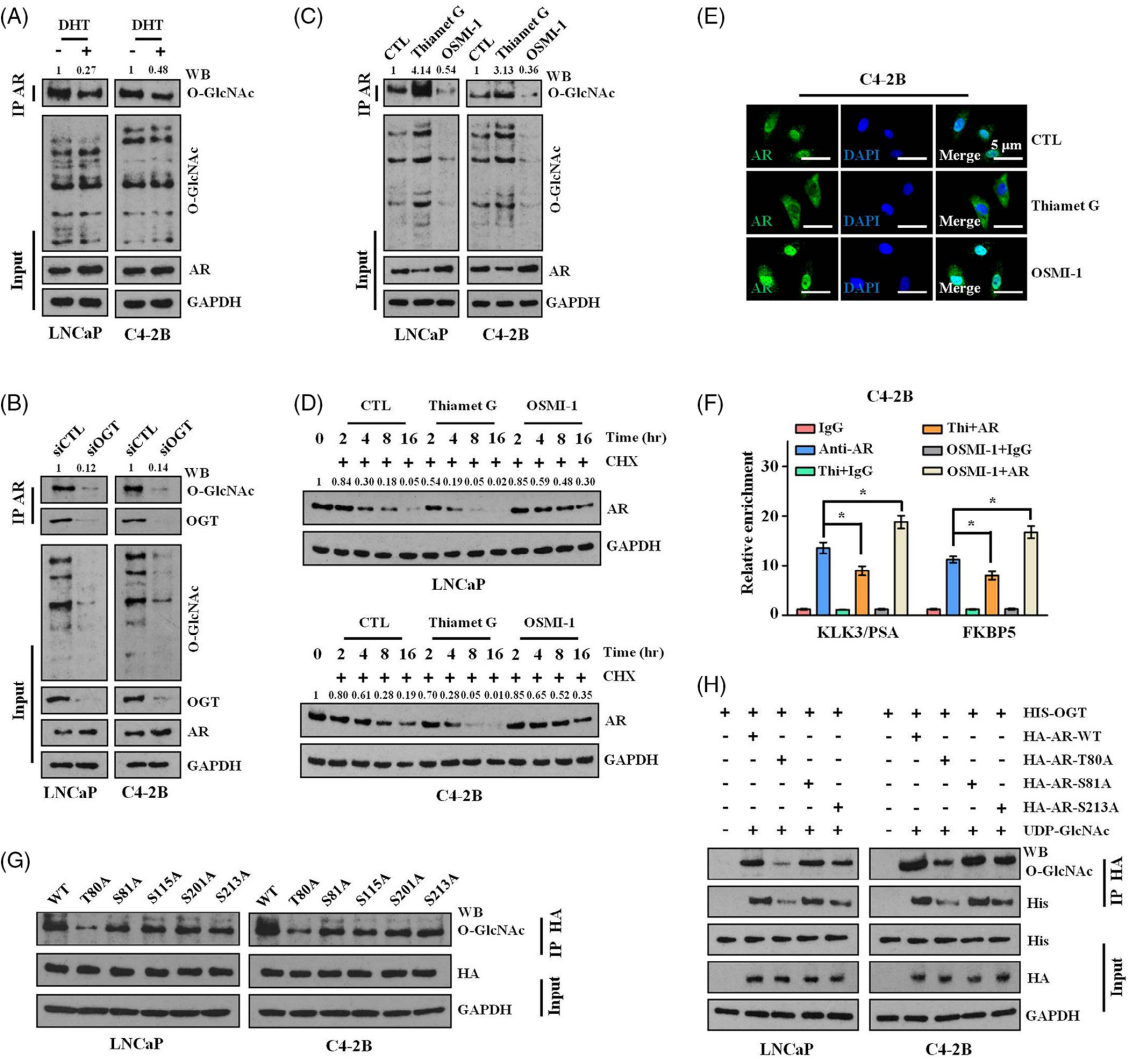
Androgen receptor (AR) threonine 80 (T80) O‐GlcNAcylation suppresses AR activation. (A–C) Immunoprecipitation of AR with anti‐AR antibody and detection of O‐GlcNAcylated protein specifically with anti‐O‐linked N‐acetylglucosamine (O‐GlcNAc) antibody were performed to analyse the O‐GlcNAcylated AR. O‐GlcNAcylated AR was examined in LNCaP and C4‐2B cells with or without (w/o) dihydrotestosterone (DHT) treatment (10 nM) (A), w/o small‐interfering RNA (siRNA) pool targeting O‐GlcNAc transferase (OGT) (B), w/o Thiamet G (25 µM) or OSMI‐1 (20 µM) treatment (C). (D) The cells were treated with cycloheximide (CHX) (10 µg/mL) for the indicated times to inhibit protein synthesis and to test stability of endogenous AR protein in LNCaP and C4‐2B cells that were cultured w/o Thiamet G (25 µM) or OSMI‐1 (20 µM). (E and F) C4‐2B cells were cultured w/o Thiamet G (25 µM) or OSMI‐1 (20 µM). Confocal microscopic images indicating the localisation of AR. Scale bar: 5 µm (E). Chromatin immunoprecipitation (ChIP)‐quantitative polymerase chain reaction (qPCR) data showing the enrichment of AR on promoter proximal regions of the KLK3 and FKBP5 genes. Data between two groups were analysed with unpaired Student's *t*‐test with ^*^ *p* < .05 (F). (G) The AR‐HA (either wild‐type [WT] or indicated mutant) expressing plasmids were transfected into LNCaP and C4‐2B cells, respectively. Exogenous AR‐HA was immunoprecipitated with an anti‐HA antibody. Co‐immunoprecipitation of O‐GlcNAc was analysed using an anti‐O‐GlcNAc antibody. (H) LNCaP and C4‐2B cells were transfected with plasmids expressing AR‐HA (either WT or indicated mutant) together with OGT‐HIS plasmid and then treated with UDP‐GlcNAc (60 mM) overnight. HA‐AR was immunoprecipitated with the anti‐HA antibody and blotted for O‐GLcNAc.

Analogous to phosphorylation, O‐GlcNAcylation is another one of the most abundant PTMs in eukaryotic cells, regulating various cellular processes, including genetic expression, cell signalling and tumourigenesis by modulating the biological function of substrate proteins.^10^, ^11^, ^12^ However, the O‐GlcNAcylation of AR, the exact sites and its impact on AR function are still not well studied. Using different online tools (YinOYang 1.2 Server, NetOGlyc 4.0 Server and GlycoMine Server), several potential O‐GlcNAc sites were consistently predicted (Figure S6A). Additionally, we successfully detected O‐GlcNAcylated proteins by western blotting in the immunoprecipitates pulled down by anti‐AR antibody in the cell lysates of LNCaP and C4‐2B (Figure 5A–C). As reported, OGT is the only known endogenous enzyme that catalyses protein O‐GlcNAcylation.^10^, ^11^, ^12^ Thus, we silenced OGT in the LNCaP and C4‐2B cells and observed that knockdown of OGT resulted in suppression of AR O‐GlcNAcylation due to the release of AR from OGT (Figure 5B). Furthermore, treating PCa cells with the O‐GlcNAcylation activator Thiamet G and the OGT inhibitor OSMI‐1, we proved that the addition of Thiamet G led to enhanced AR O‐GlcNAcylation, while OSMI‐1 induced a similar inhibitory effect as OGT knockdown (Figure 5C). Collectively, these results indicate that AR protein is O‐GlcNAcylated in PCa cells.

Intriguingly, in contrast to stimulating AR phosphorylation, the addition of DHT attenuated AR O‐GlcNAcylation (Figure 5A). As mentioned earlier, AR S81 phosphorylation, which results from androgen supplementation, contributes to AR transactivation. Can AR O‐GlcNAcylation affect AR action? We observed that OSMI‐1 protected the AR protein from degradation, while Thiamet G accelerated AR degradation in CHX chase experiments (Figure 5D). Based on IF and ChIP‐qPCR assays for AR protein, we further found that OSMI‐1 triggered AR nuclear translocation and AR occupancy on the promoter proximal regions of AR‐targeted genes, including KLK3 and FKBP5, whereas Thiamet G induced inverse effects on AR localisation (Figure 5E,F). Furthermore, Thiamet G significantly repressed the proliferation, invasion and migration of C4‐2B cells, while OGT silencing or use of its inhibitor moderately enhanced the aggressiveness of C4‐2B under the same conditions (Figure S6B–G). Based on these findings and the fact that Thiamet G, OSMI‐1 and OGT knockdown can modulate the level of AR O‐GlcNAcylation, we concluded that AR O‐GlcNAcylation may exert an inhibitory effect on AR transactivation in PCa cells.

Five amino acids in AR, namely, T80, S81, S115, S201 and S213, were consistently predicted to be directly modified by O‐GlcNAcylation based on online tools (Figure S6A). To confirm the exact sites of O‐GlcNAcylation modification, we performed point mutation experiments for these amino acids within AR. We observed that the levels of O‐GlcNAc‐modified AR were almost equal to WT AR when S81, S115, S201 and S213 were mutated. However, when T80 was mutated, the levels of O‐GlcNAc‐modified AR were notably decreased (Figure 5G). This finding suggests that T80 is one of the O‐GlcNAc sites within this peptide. Furthermore, we performed exogenous O‐GlcNAcylation experiments by overexpressing HIS‐tagged OGT and HA‐tagged WT/mutated AR proteins. These experiments resulted in potent O‐GlcNAcylation of exogenous AR proteins except for those with the T80A mutation. We also found that the T80A mutation impaired the binding of the AR protein with exogenous OGT (Figure 5H). Based on these data, we conclude that the AR protein can be O‐GlcNAcylated at T80 by OGT.

### 3.6 Mutual antagonism between AR phosphorylation at S81 and AR O‐GlcNAcylation at T80

**FIGURE 6:**
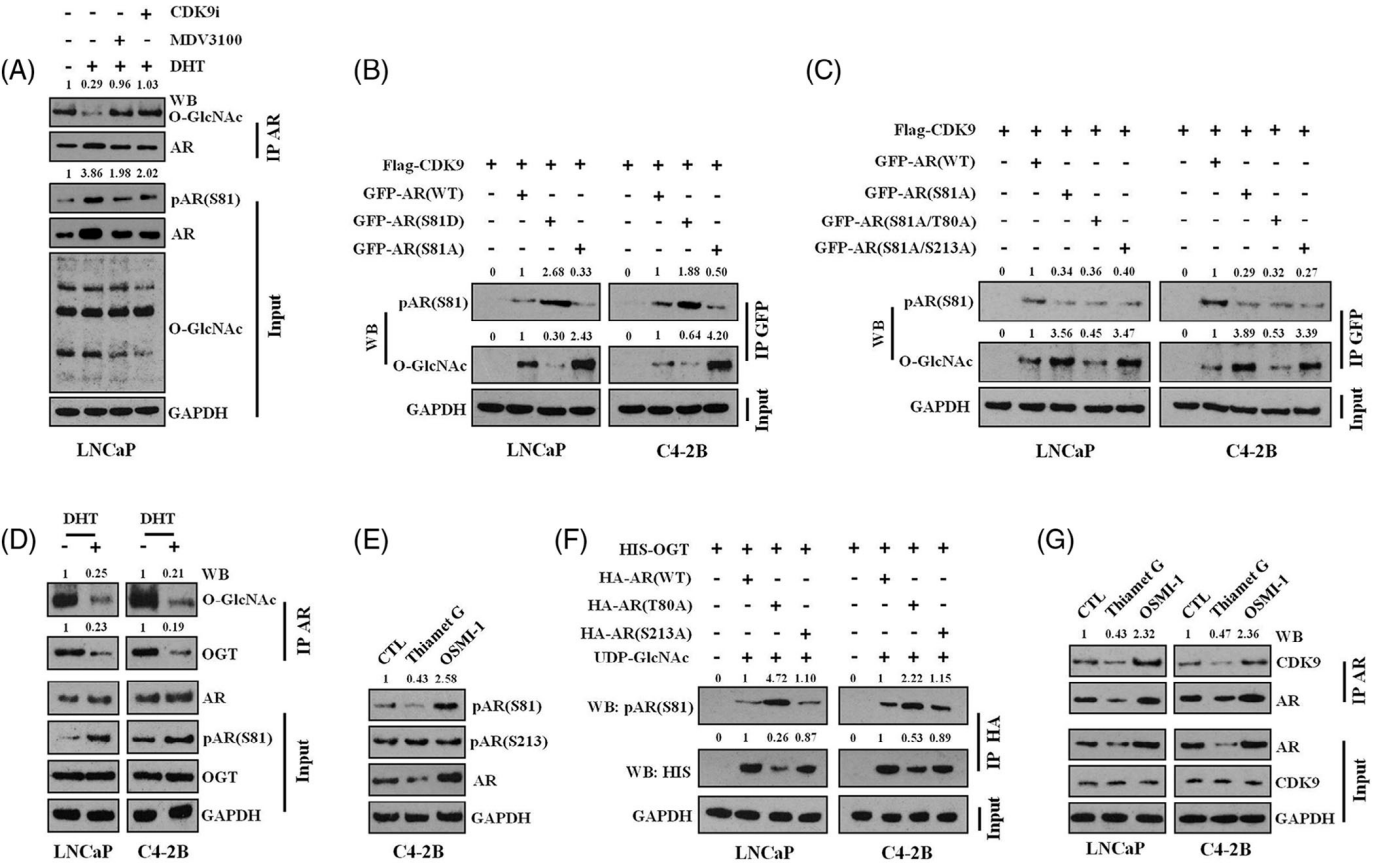
Androgen receptor (AR) phosphorylation and AR O‐GlcNAcylation are mutually antagonised. (A) LNCaP cells were treated with or without (w/o) dihydrotestosterone (DHT) (10 nM), MDV3100 (10 µM) and the CDK9 inhibitor BAY1251152 (10 µg/mL) for 24 h. O‐GlcNAcylated AR was determined as described above. Immunoblotting assay was performed using whole cell lysis to measure the AR, phosphorylated AR serine 81 (S81) and global O‐GlcNAcylation (O‐linked N‐acetylglucosamine [O‐GlcNAc]). (B and C) LNCaP and C4‐2B cells were transfected with plasmids expressing AR‐GFP (either wild‐type [WT] or indicated mutant) together with CDK9‐Flag plasmid. GFP‐AR was immunoprecipitated with the anti‐green fluorescent protein (GFP) antibody and blotted for O‐GlcNAc and AR S81 phosphorylation. (D) LNCaP and C4‐2B cells were treated w/o DHT (10 nM) for 24 h. AR was immunoprecipitated with the anti‐AR antibody and blotted for O‐GlcNAc and O‐GlcNAc transferase (OGT). Immunoblotting was performed using whole cell lysis to determine the levels of AR, phosphorylated AR S81 and OGT. (E) C4‐2B cells were cultured w/o Thiamet G (25 µM) or OSMI‐1 (20 µM) for 24 h. The levels of AR, AR S81 phosphorylation and AR S213 phosphorylation as determined by immunoblotting. (F) LNCaP and C4‐2B cells were transfected with plasmids expressing AR‐HA (either WT or indicated mutant) together with OGT‐HIS plasmid and then treated with UDP‐GlcNAc (60 mM) overnight. HA‐AR was immunoprecipitated with the anti‐HA antibody and blotted to measure the expression level of HIS‐OGT and phosphorylated AR S81. (G) LNCaP and C4‐2B cells were cultured w/o Thiamet G (25 µM) or OSMI‐1 (20 µM) for 24 h. AR was immunoprecipitated with the anti‐AR antibody and blotted against CDK9 and AR. The expression levels of AR and CDK9 were also detected by immunoblotting in whole cell lysis.

In some cases, phosphorylation and O‐GlcNAcylation exhibit mutual antagonism, which is critical for protein stability and activity.^12^, ^13^ Given that phosphorylation at S81 and O‐GlcNAcylation at the nearby T80 site within the AR protein have opposite effects on AR function, we hypothesised that they might be reciprocally regulated. To test this, we examined their changes in expression under various conditions, including the addition of androgen, AR antagonists and CDK9 inhibitor. Interestingly, we found that alterations in AR S81 phosphorylation expression were accompanied by corresponding changes in AR O‐GlcNAcylation at T80 in opposite directions (Figure 6A). Moreover, we expressed exogenous GFP‐tagged AR, either WT or with S81D or S81A mutations, along with exogenous CDK9 in LNCaP and C4‐2 cells. We observed that O‐GlcNAcylation of AR was more abundant in the S81A mutant, but notably repressed in the S81D mutant, compared to WT AR (Figure 6B). To investigate whether the level of AR O‐GlcNAcylation level is determined by O‐GlcNAcylation at the T80 site, we transfected exogenous AR with S81A/T80A or S81A/S213A mutations into PCa cells under similar conditions. We found that the additional mutation in the T80 site diminished AR O‐GlcNAcylation, while the additional mutation in the S213 site had no impact on AR O‐GlcNAcylation (Figure 6C). AR‐V7 is an alternatively spliced variant of AR that lacks the functional ligand‐binding domain but still contains complete DNA‐binding domain and NTD domain.

Therefore, AR‐V7 is constitutively active without the need for ligand binding.^3^ We then expressed exogenous GFP‐tagged AR‐V7, either WT or with S81D or S81A mutations in AR‐V7‐null LNCaP cells. As expected, WT AR‐V7 was notably phosphorylated in the S81 site. Moreover, O‐GlcNAcylation of AR was also more abundant in the S81A mutant, compared to WT AR‐V7 and the S81D mutant (Figure S7A). Further experiments in the same setting using GFP‐AR‐V7 vectors comprising double mutations also verified that the additional mutation in the T80 site diminished AR O‐GlcNAcylation in the S81A mutant (Figure S7B). These data collectively suggested that phosphorylation at S81 antagonises AR O‐GlcNAcylation at the T80 site. This was further confirmed by our finding that DHT treatment simulated AR S81 phosphorylation, but meanwhile attenuated the interaction of OGT with AR, leading to a decreased AR O‐GlcNAcylation (Figure 6D).

Furthermore, we have observed that stimulation or inhibition of cellular O‐GlcNAcylation can specifically reduce or elevate AR S81 phosphorylation (Figure 6E). Based on this, we hypothesised that O‐GlcNAcylation of AR at T80 might be responsible for this effect. To test this possibility, we transfected exogenous HA‐tagged AR, WT or T80A, or S213A mutant, into PCa cells in experiments designed to modulate exogenous O‐GlcNAcylation. We found that phosphorylation of AR at S81 was more abundant in the T80A mutant, but not in the S213A mutant, compared to WT AR (Figure 6F), indicating that O‐GlcNAcylation at T80 within the AR protein inhibits phosphorylation at S81. This phenomenon may be explained by the decreased AR‐CDK9 binding due to AR O‐GlcNAcylation (Figure 6G). Collectively, these results establish a close relationship that was mutually exclusive between phosphorylation at S81 and O‐GlcNAcylation at T80 within the AR protein.

### 3.7 LINC01126 promotes the switch from O‐GlcNAcylation to phosphorylation within the AR protein

**FIGURE 7:**
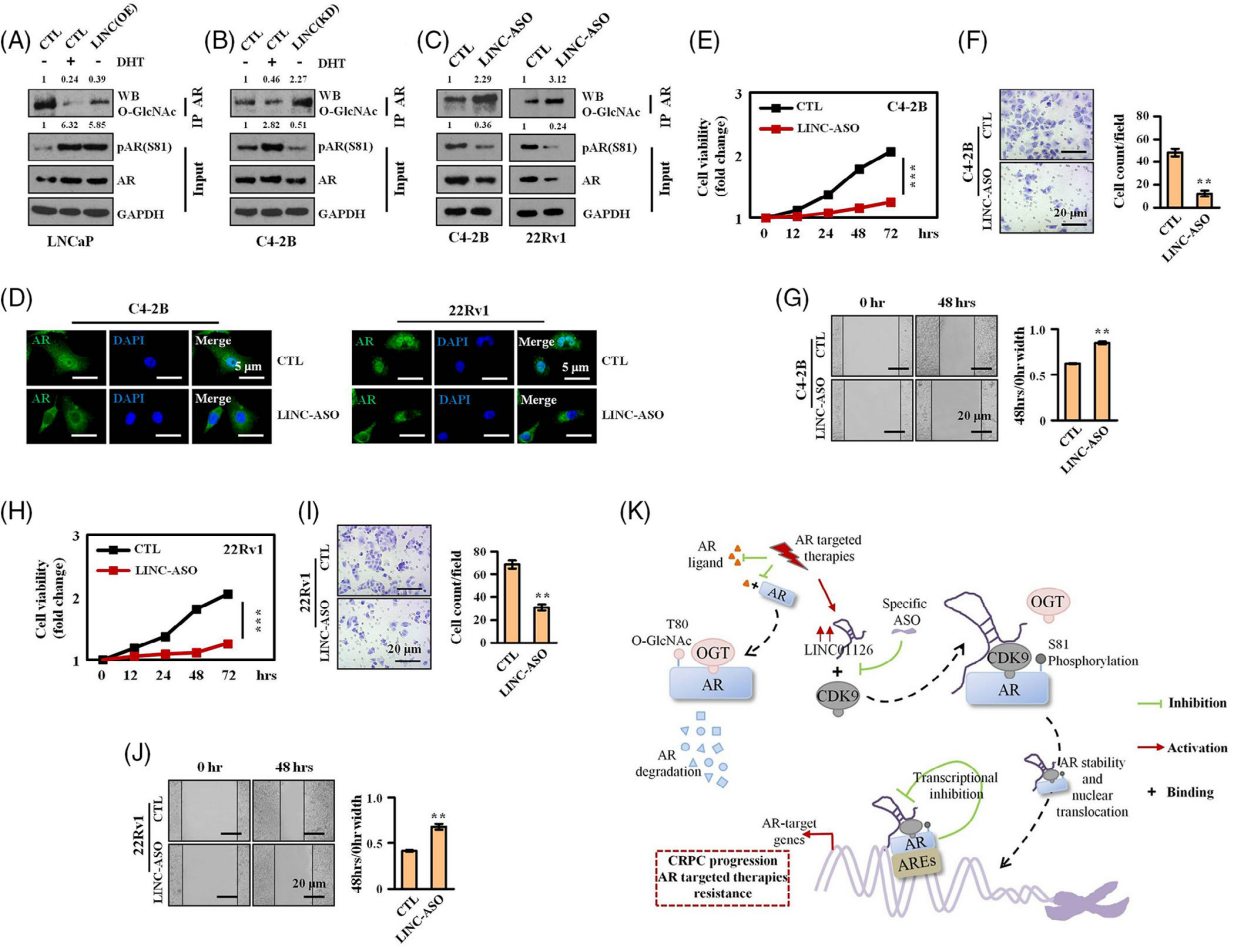
LINC01126 drives the switch from androgen receptor (AR) O‐GlcNAcylation to AR phosphorylation. (A and B) The level of O‐GlcNAcylated AR was determined as described in above and examined in LNCaP cells with or without (w/o) dihydrotestosterone (DHT) treatment (10 nM) and LINC01126 overexpression (A), and in C4‐2B cells w/o DHT treatment (10 nM) and LINC01126 knockdown (B). (C) O‐GlcNAcylated AR was examined in castration‐resistant prostate cancers (CRPC) C4‐2B cells and 22Rv1 cells w/o antisense oligos (ASO) targeting LINC01126. AR and AR serine 81 (S81) phosphorylation level were also detected by immunoblotting in whole cell lysis (A–C). (D–J) C4‐2B and 22RV1 cells w/o ASO targeting LINC01126 were cultured in androgen‐deprived medium. (D) Confocal microscopic images indicating the localisation of AR. Scale bar: 5 µm. (E and H) cell counting kit‐8(CCK‐8) cell viability assays. (F and I) Representative images and quantification of the invaded cells derived from transwell assays. Scale bar: 20 µm. (G and J) Representative images and quantification of cell migration derived from wound healing assays. Scale bar: 20 µm. (K) Schematic illustration of the function of LINC01126 in CRPC progression. AR‐targeted therapies inhibit synthesis of AR ligand or block binding of AR ligand to AR. Without AR ligand, AR protein is O‐GlcNAcylated at threonine 80 and degrades rapidly. LINC01126 is transcriptionally repressed by AR. AR‐targeted therapies thereby upregulate LINC01126 expression through relieving its transcriptional repression by AR. LINC01126 facilitates the interaction of AR with CDK9, resulting in AR S81 phosphorylation. Consequences of AR S81 phosphorylation are analogous to the effect of androgen on AR stability, nuclear translocation and transactivation. Therefore, LINC01126 facilitates the activation of AR signalling independent of androgen, contributing to progression of CRPC. LINC01126‐ASO that specifically interferes the binding of AR to CDK9 may have a possible therapeutic effect on CRPC. AREs, androgen‐responsive elements. Data between two groups were analysed with unpaired Student's *t*‐test with ^**^ *p* < .01 and ^***^ *p* < .001.

We have previously described how LINC01126 enhances the phosphorylation of AR at S81 by facilitating the interaction of AR with CDK9. Additionally, phosphorylation of AR at S81 antagonises O‐GlcNAcylation of AR at T80. However, it remains unclear whether LINC01126 can trigger the switch from O‐GlcNAcylation to phosphorylation of AR in PCa cells. To investigate this, we performed Co‐IP experiments and obtained immunoprecipitates using anti‐AR antibodies in PCa cells. Our results indicate that LINC01126 overexpression attenuates the O‐GlcNAcylation of AR while enhancing AR S81 phosphorylation, whereas LINC01126 knockdown induces an inverse change in AR PTMs (Figure 7A,B). We also found that LINC01126‐ASO inhibits the CDK9 interaction region within LINC01126, leading to a repression of AR S81 phosphorylation. To further explore the effect of LINC01126‐ASO on AR O‐GlcNAcylation, we performed Co‐IP experiments. Our results show that LINC01126‐ASO effectively promotes the level of AR O‐GlcNAcylation but inhibits AR S81 phosphorylation in CRPC C4‐2 and 22Rv1 cells (Figure 7C).

We have previously shown that LINC01126‐mediated AR transactivation promotes the progression of PCa cells. Based on these findings, we attempted to determine the therapeutic potential of LINC01126‐ASO in restricting CRPC cells. We performed IF experiments in CRPC C4‐2B and 22RV1 cells and found that LINC01126‐ASO promoted the escape of AR proteins from the nucleus, indicating that LINC01126‐ASO inhibits AR transactivation (Figure 7D). Additionally, cell proliferation, migration and invasion abilities were significantly impaired after LINC01126 interference by ASO compared to the negative control‐ASO group (Figure 7E–J). These results collectively testify that LINC01126 plays a critical role in the switch from O‐GlcNAcylation to phosphorylation within the AR protein and that targeting LINC01126 with ASO may serve as a promising therapeutic approach for CRPC.

## 4 DISCUSSION

Although CRPC usually emerges following AR‐targeted therapies, including ADT and ARPIs, the majority of CRPC tumours still rely on an enhanced AR protein level or signalling.^3^ This fact supports the notion that other players, besides AR itself, may participate in CRPC pathogenesis. Accumulating evidence indicates that lncRNAs play important roles in the progression of CRPC.^15^ In this study, we present a novel working model wherein the lncRNA LNC01126 facilitates the binding of CDK9 but impairs the binding of OGT to the AR protein, which leads to the transition from O‐GlcNAcylation at AR T80 to phosphorylation at AR S81. These alterations in PTMs of the AR protein prevent AR protein degradation, promote AR nuclear translocation, and enhance the expression of AR‐activated genes. These findings reveal a distinctive mode of operation of lncRNAs as protein scaffolds in maintaining sustained AR signalling at the post‐translational level during CRPC progression (Figure 7K). The mechanism we identified echoes previous reports on lncRNAs such as HOTAIR,^27^ PCBP1‐AS1^28^ and KDM4A‐AS1,^29^ which emphasises the role of lncRNAs in regulating the PTM profile of the AR protein. Furthermore, lncRNAs may support the AR‐mediated program through other mechanisms^18^, ^19^: (1) stabilising AR mRNA, (2) regulating AR mRNA splicing, (3) promoting AR translation and (4) facilitating AR transactivation.

As the most frequently observed PTMs with highly reversible and dynamic features, phosphorylation and O‐GlcNAcylation cooperatively participate in modulating protein functions under different biological contexts.^12^, ^13^ The phosphorylation sites at the AR protein and their functional consequences have already been well documented, among which S81 phosphorylation represents a classic model in which the AR protein can be phosphorylated by androgens and/or kinases from the CDK family, leading to transactivation.^5^, ^30^ Here, we propose that in the milieu of low androgen or when AR is antagonised by ARPIs (e.g., Enza), LINC01126 can replace androgen to facilitate CDK9 interaction with AR and maintain AR signalling, which is required for CRPC establishment.

Aside from phosphorylation, the impact of O‐GlcNAcylation on PCa progression has been gradually explored and is still debated. Li et al. reported that OGT‐mediated O‐GlcNAcylation stabilises Bmi‐1 protein, resulting in stem cell self‐renewal and tumourigenesis initiation in PCa.^31^ Several studies that focused on OGT and O‐GlcNAcylation at the cellular level have also supported their oncogenic role in PCa.^32^, ^33^, ^34^ However, it is worth noting that most of these studies employed hormone‐sensitive PCa tissues and AR‐null PCa cells as research models, which may have caused selection bias. On the other hand, Sayat et al. reported that O‐GlcNAcylation of beta‐catenin, a TF that plays a critical role in CRPC progression, attenuates its nuclear localisation and transcriptional activity.^35^, ^36^ Another team declared that repression of the hexosamine biosynthetic pathway, which produces UDP‐GlcNAc that serves as substrate for O‐GlcNAcylation, promotes CRPC establishment,^37^ indicating a suppressive role of O‐GlcNAcylation in CRPC. However, based on our best knowledge, none of the previous studies investigated the O‐GlcNAcylation of the AR protein. The present study confirmed for the first time that AR can be O‐GlcNAcylated at T80, which leads to attenuated AR‐mediated signals. Moreover, AR T80 O‐GlcNAcylation and S81 phosphorylation are mutually exclusive. This mode of action, through which O‐GlcNAcylation competes with phosphorylation at the same or nearby sites to regulate TFs, was reported in PCa for the first time. Consistently, some other cases with the interplay between the two types of PTMs have already been uncovered in various chronic diseases such as sexual dysfunction, cardiovascular disease, neurodegenerative disease and cancer.^38^, ^39^ Interestingly, a recent study demonstrated that inhibition of CDK9 leads to OGT‐dependent proteome remodelling in PCa, further supporting their functional exclusion.^40^ In summary, further efforts are needed to elucidate the significance of O‐GlcNAcylation in PCa progression and AR signals.

In this study, we confirmed that LINC01126 is an androgen‐repressed gene that is transcriptionally repressed by AR. AR antagonist can competitively bind with the ligand‐binding domain of AR protein and thus block the action of androgen. Then, the repression of LINC01126 is relieved, which may combine with other factors (e.g., activating TFs and epigenetic modifications) to facilitate the promotion of LINC01126 expression. More and more evidence supports the notion that AR‐targeted therapies drive the formation of CRPC.^3^ Accompany with the progression of CRPC, the level of LINC01126 may be chronically elevated, which contributes to enhanced AR‐mediated gene expression program in CRPC tumours. It is easy to understand the continued expression of AR‐activated genes in CRPC tumours with high AR activity. For instance, a positive feedback loop between lncRNA ARLNC1 and the AR protein has been well established, wherein ARLNC1 stabilises AR transcripts to strengthen AR protein levels, and AR, in turn, transcriptionally activates ARLNC1 expression.^17^ However, the question remains: why are androgen‐repressed genes such as LINC01126 able to avoid AR‐mediated repression in similar conditions? A similar case can be seen in the lncRNA HOTAIR, which is also an AR‐repressed gene that prevents AR protein from undergoing ubiquitin‐mediated degradation in CRPC tumours but avoids being repressed.^27^ Interestingly, AR itself is androgen‐repressed.^41^ As reported, there is an enhancer in the second intron of the AR gene that mediates elevated AR expression in castration settings with a low androgen level.^41^ Moreover, the distinct epigenetic modifications around the enhancer in castrated androgen levels also contribute to the elevated AR expression.^41^ Therefore, we postulate that the expression of LINC01126 and HOTAIR may be increased in CRPC cells by similar mechanisms.

Although we have presented a rational explanation for sustained AR signals in CRPC, this is still a preliminary study with several limitations. First, the reason why the interaction of CDK9 with AR protein impairs the binding of OGT with AR protein is still unclear. As T80 and S81 are neighbouring sites on the AR protein, CDK9 and OGT may compete for the same structure on the AR protein for docking. Second, the biological efficacy and safety of LINC01126‐ASO should be fully evaluated in in vivo experiments to exclude possible adverse reactions. Furthermore, the regulation of AR PTMs by LINC01126 may only be one of the multiple mechanisms underlying CRPC formation. It is worthwhile to explore whether LINC01126 can function through an AR‐independent mechanism in the future. Lastly, this study mainly explored WT AR, whether LINC00126 can regulate other AR splicing variants (e.g., AR‐V7, AR‐V567es) that comprising complete NTD warrants further research.

## 5 CONCLUSIONS

In summary, our study has revealed a novel role for LINC01126 in promoting the castration resistance of PCa by enhancing AR‐mediated transactivation through modulating PTMs of AR protein. Thus, LINC01126 may serve as a prognostic marker for PCa and a promising therapeutic target for CPRC.

## AUTHOR CONTRIBUTIONS

*Conception and design*: Yu Gan, Yi Cai, Minfeng Chen and Zhi Long. *Collection data from public databases and bioinformatic analyses*: Yu Gan, Yuchen Gong and Zhiwei Shu. *Collection surgical specimens*: Zhiwei Shu, Guyu Tang and Yao He. *Experimental works*: Yu Gan, Yuchen Gong, Hengfeng Zhou, Jiaxian Chen and Guyu Tang. *Manuscript writing*: Yi Cai and Yu Gan. *Final approval of manuscript*: all authors.

## CONFLICT OF INTEREST STATEMENT

The authors declare they have no conflicts of interest.

## ETHICS STATEMENT

This study was approved by the Research Ethics Committee of Xiangya Hospital of CSU (2021101079), and informed consent was obtained from each patient. The animal procedures were conducted according to the standards of the National Institutes of Health Guide for the Care and Use of Laboratory Animals and approved by the CSU Animal Ethics Committee (2020sydw0041).

## Supporting information

Supporting Information

Supporting Information

## Data Availability

The datasets used and/or analysed during the current study are available from the corresponding author upon reasonable request.
