# Supplementary material for: Androgen‐repressed lncRNA LINC01126 drives castration‐resistant prostate cancer by regulating the switch between O‐GlcNAcylation and phosphorylation of androgen receptor
Source: Clin Transl Med. 2024 Jan 12;14(1):e1531. doi: 10.1002/ctm2.1531 (PMC10785194; doi:10.1002/ctm2.1531)
Supplement: Supplementary file 2 — Supporting Information [file CTM2-14-e1531-s002.docx]

# Androgen-repressed lncRNA LINC01126 drives castration-resistant prostate cancer by regulating the switch between O-GlcNAcylation and phosphorylation of androgen receptor

Running title: Gain of LINC01126 in prostate cancer

Yi Cai^1*^, Minfeng Chen^1*^, Yuchen Gong^1^, Guyu Tang^1^, Zhiwei Shu^1^, Jiaxian Chen1,

Hengfeng Zhou^2^, Yao He^1^, Zhi Long^2^, Yu Gan^1^

1. Department of Urology, Disorders of Prostate Cancer Multidisciplinary Team, National Clinical Research Center for Geriatric Disorders, Xiangya Hospital, Central South University, Changsha City, Hunan Province, P.R. China, 410008;
2. Andrology Center, Department of Urology, the Third Xiangya Hospital, Central South University, Changsha City, Hunan Province, P.R. China, 410013;

* YC and MC contributed equally to this work.

**Corresponding author:** Yu Gan, M.D.

No. 87 Xiangya Road, Changsha City Hunan Province, P.R. China, 410008 Tel: +86 15111140206

[Email: ganyu@csu.edu.cn](mailto:ganyu@csu.edu.cn)

**Disclosure Statement:** The authors have declared that no conflict of interest exists.

# Supplementary Materials

- 1. **RNA and Plasmid Information**
  2. **Antibody Information**
  3. **Primers for qPCR**
  4. **Supplementary Figures**

**Supplementary Tables**

**Table S1. List of 47 genes that were consistently upregulated in metastatic tumors in three clinical prostate cancer cohorts (GSE8511, MCTP cohort and MSKCC cohort).**

**Table S2. List of 350 genes that were directly repressed by the AR (reported by Zhang YJ and colleagues PMID: 29808028)**

**Table S3. GSEA analysis_MSKCC (High LINC01126 versus Low LINC01126)**

**Table S4. GSEA analysis_MCTP (High LINC01126 versus Low LINC01126)**

**Table S5. Top 10 genes with hihg scores in mass spectrometry (MS)**

**Ⅰ. RNA and Plasmid Information**

| **Reagent** | **Provider** | **Catalogue#/Sequence** | |
| --- | --- | --- | --- |
| ON-TARGETplus Human  OGT siRNA | Dharmacon | L-019111-00-0005 | |
| ON-TARGETplus Human  AR siRNA | Dharmacon | L-003400-00-0005 | |
| ON-TARGETplus Human  CDK9 siRNA | Dharmacon | L-003243-00-0005 | |
| [ON-TARGETplus](https://horizondiscovery.com/en/products/gene-modulation/knockdown-reagents/controls/PIFs/ON-TARGETplus-Non-targeting-Control-Pool?catalognumber=D-001810-10-05)  [Non-targeting Pool](https://horizondiscovery.com/en/products/gene-modulation/knockdown-reagents/controls/PIFs/ON-TARGETplus-Non-targeting-Control-Pool?catalognumber=D-001810-10-05) | Dharmacon | D-001810-10-05 | |
| LINC01126-shRNA1 | Origene | F: | ATGTGATTTGCCTAAGACCTT |
|  |  | R: | GGTCTTAGGCAAATCACATTT |
| LINC01126-shRNA2 |  | F: | TGAAATCTTGTTACTAACCTT |
|  |  | R: | GGTTAGTAACAAGATTTCATT |
| LINC01126-shRNA3 |  | F: | ATTAGACATTTTATACTAGTT |
|  |  | R: | CTAGTATAAAATGTCTAATTT |
| Control shRNA |  | F: | TTCTCCGAACGTGTCACGTTT |
|  |  | R: | ACGTGACACGTTCGGAGAATT |
| Flag-CDK9 | Addgene | #28100, pCMV-hAR was a gift from [Andrew Rice](https://www.addgene.org/Andrew_Rice/). | |
| His-OGT | GenePharma | #100135A | |
| GFP-AR-WT/MT |  | #100165 | |
| GFP-AR-V7-WT/MT |  |  |  |
| HA-AR-WT/MT |  |  |  |
| pGL3-Promoter Vector | Promega | E1761 | |
| pGL3-KLK3 ARE(+) | GenePharma | #100129 | |
| pGL3-KLK3 ARE(-) |  |  |  |
| DIG-labeled LINC01126 probe for ISH | Boster | 5′-GATGTGATTTGCCTAAGACCAC ATGATTTCTCTGGGGCA-3′ | |
| Control probe for ISH | Boster | 5′-GTGTAACACGTCTATACGCCCA-  3′ | |
| LINC01126 FISH probes | Ribo Bio | 5′-TGTGATGTGATTAGCAAAGCCA GC-3′ | |
|  | Ribo Bio | 5′-ACAAGGAAACCCAAGTACCAA  AGAG-3′ | |
|  | Ribo Bio | 5′-CACCAAAGAACAAACCAGACC AC-3′ | |
| CY3-labeled U6 | Ribo Bio | 5′-CACGAATTTGCGTGTCATCCTT-  3′ | |
| LINC01126-ASO | GenePharma | 5′-AGTCAGCTCCCCCGCCCGGGAC  GTCCCGCGCCACTCCGCGCCTTTG GCCCTGGCTCAAGGTCTTGTGA-3′ | |
| LINC01126-ASO(mutated) |  | 5′-CCGAGGCTCCCGGATCCGGGTT  GCCCCGCGCCCGAATGCGCCTAACGCCCCCAGTCAAGGTCTGCCGT-3′ | |
| LINC01126-ASO(non-targeted) |  | 5′-AGCCAGCGCCTTGTCCTCAGAC  ACTCAGCCCTGCCCGGCAGGCCCCGGCGCTCAAGCCCTGTTTACTGAGCC-3′ | |

**Ⅱ. Antibody Information**

| **Antibody** | **Vendor** | **Catalogue**  **Number** | **Application** | **Dilution** |
| --- | --- | --- | --- | --- |
| AR | Invitrogen | MA5-13426 | WB | 1:1000 |
|  |  |  | IP | 1:50 |
|  |  |  | IF | 1:200 |
|  |  |  | IHC | 1:200 |
| PSA | Abcam | ab53774 | WB | 1:1000 |
|  |  |  | IHC | 1:200 |
| Ki-67 | Proteintech | 27309-1-AP | IHC | 1:1000 |
| CDK1 | Proteintech | 19532-1-AP | WB | 1:1000 |
| CDK2 | Proteintech | 10122-1-AP | WB | 1:500 |
| CDK5 | Proteintech | 10430-1-AP | WB | 1:500 |
| CDK7 | Proteintech | 27027-1-AP | WB | 1:1000 |
| CDK9 | Abcam | ab239364 | WB | 1:1000 |
|  |  |  | IP | 1:50 |
| Histone H3 | CST | 4499 | WB | 1:1000 |
| GAPDH | Abcam | ab125247 | WB | 1:2000 |
| O-GlcNAc | Abcam | ab2739 | WB | 1:300 |
| OGT | Proteintech | 11576-2-AP | WB | 1:1000 |
| AR(S81) | Millipore | 04-078 | WB | 1:1000 |
|  |  |  | IHC | 1:200 |
| AR(S94) | Invitrogen | PA5-35386 | WB | 1:1000 |
| AR(S210) AR(S213) | Invitrogen | MA5-16221 | WB | 1:500 |
| AR(Y534) | Invitrogen | PA5-105959 | WB | 1:1000 |
| AR(Y267) | Invitrogen | PA5-105946 | WB | 1:1000 |
| Vinculin | Sigma Aldrich | V9131-2ML | WB | 1:2000 |
| 6X HIS | Abcam | ab18184 | WB | 1:500 |
| GFP | Abcam | ab290 | WB | 1:1000 |
|  |  |  | IP | 1:50 |
| Flag | Abcam | ab205606 | WB | 1:1000 |
|  |  |  | IP | 1:50 |
| HA | Proteintech | 51064-2-AP | WB | 1:3000 |
|  |  |  | IP | 1:50 |

**Ⅲ. Primers for qPCR**

| **Primer name** | **Forward Primer Sequence (5’-3’)** | **Reverse Primer Sequence (5’-3’)** |
| --- | --- | --- |
| LINC01126 | GCAACTGTGGTCTGGTTTGT | TGTCCCGTAACATGTCCACA |
| FKBP5 | TTCTCTGTTCTGGCGTGAGT | GGTTCCTTGGGTTGCCAAAT |
| KLK3 | AGTGCGAGAAGCATTCCCAAC | CCAGCAAGATCACGCTTTTGTT |
| AR | CCAGGGACCATGTTTTGCC | CGAAGACGACAAGATGGACAA |
| CDK1 | AAACTACAGGTCAAGTGGTAGCC | TCCTGCATAAGCACATCCTGA |
| CDK2 | CCAGGAGTTACTTCTATGCCTGA | TTCATCCAGGGGAGGTACAAC |
| CDK5 | GGAAGGCACCTACGGAACTG | GGCACACCCTCATCATCGT |
| CDK9 | TTGCGGGAGATCAAGATCCTT | TACCCTTGCAGCGGTTATAGG |
| 18s | CAGCCACCCGAGATTGAGCA | TAGTAGCGACGGGCGGTGTG |
| U1 | GGGAGATACCATGATCACGAAGT | CCACAAATTATGCAGTCGAGTTTCCC |
| GAPDH | GGAGCGAGATCCCTCCAAAAT | GGCTGTTGTCATACTTCTCATGG |
| LINC-P1 ChIP | TCCAAGAACTACAACGGCTG | CTCGCCCGTTATTCGTCGT |
| LINC-P2 ChIP | AAGGAGAGAAGCGAGGAGC | ACAGGGCAGGGAAAGACAC |
| LINC-P3 ChIP | GAATATCGCAACCATCCCCG | AAGCCCTGTTTACTGAGCCT |
| FKBP5  ChIP | AAGCCACTAGTTACGCCACT | GGAGAGGCTGACATCCAAGT |
| KLK3 ChIP | CTGGATTCTGGGTTGGGAGT | CACGTGCCTAGATCCTTTGC |
| GAPDH ChIP | TACTAGCGGTTTTACGGGCG | TCGAACAGGAGGAGCAGAGAGCGA |

**IV. Supplementary Figures**

**Supplementary Figure 1**

**
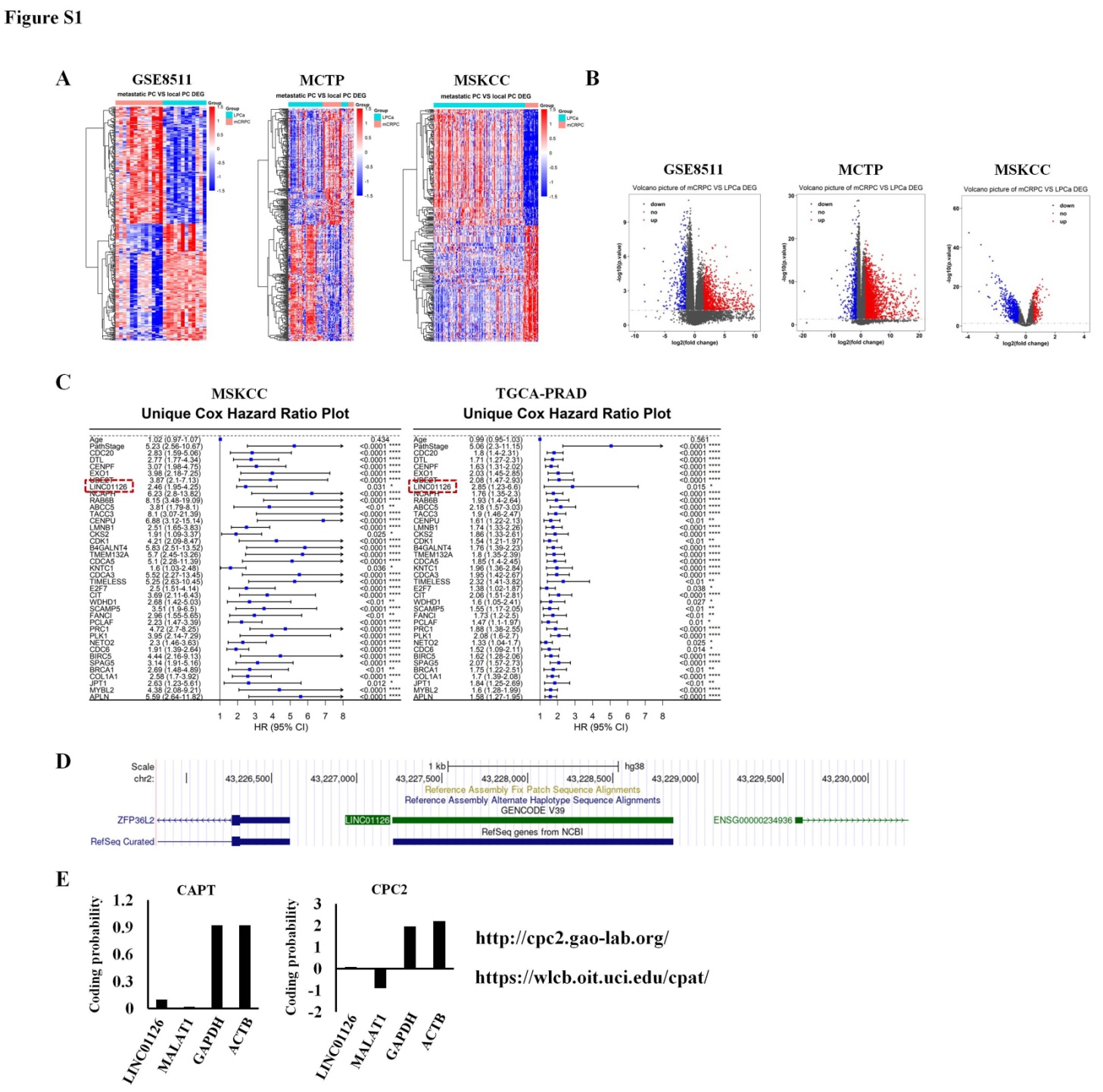
**

**Figure S1. (A)** Heatmaps of top 400 DEGs compared metastatic tumors with localized tumors in three published clinical PCa cohorts (GSE8511, MCTP cohort, and MSKCC cohort). **(B)** Volcano pictures of DEGs compared metastatic tumors with localized tumors in the same cohorts as **(A)**. **(C)** Thirty-seven genes from the genes consistently upregulated in metastatic tumors in the cohorts mentioned above were validated in MSKCC cohort and TGCA-PRAD cohort to increase the risk of biological recurrence (BCR) by univariate Cox regression analysis. **(D)** Schematic diagram of the genomic locus and transcript of LINC01126 in the UCSC Genome Browser [(http://genome.ucsc.edu/).](http://genome.ucsc.edu/)) **(E)** The coding potential of LINC01126 as predicted by the CPAT and CPC2 tools. The lncRNA MALAT1 and the protein-coding genes GAPDH and ACTB are also shown as references.

**Supplementary Figure 2**

**
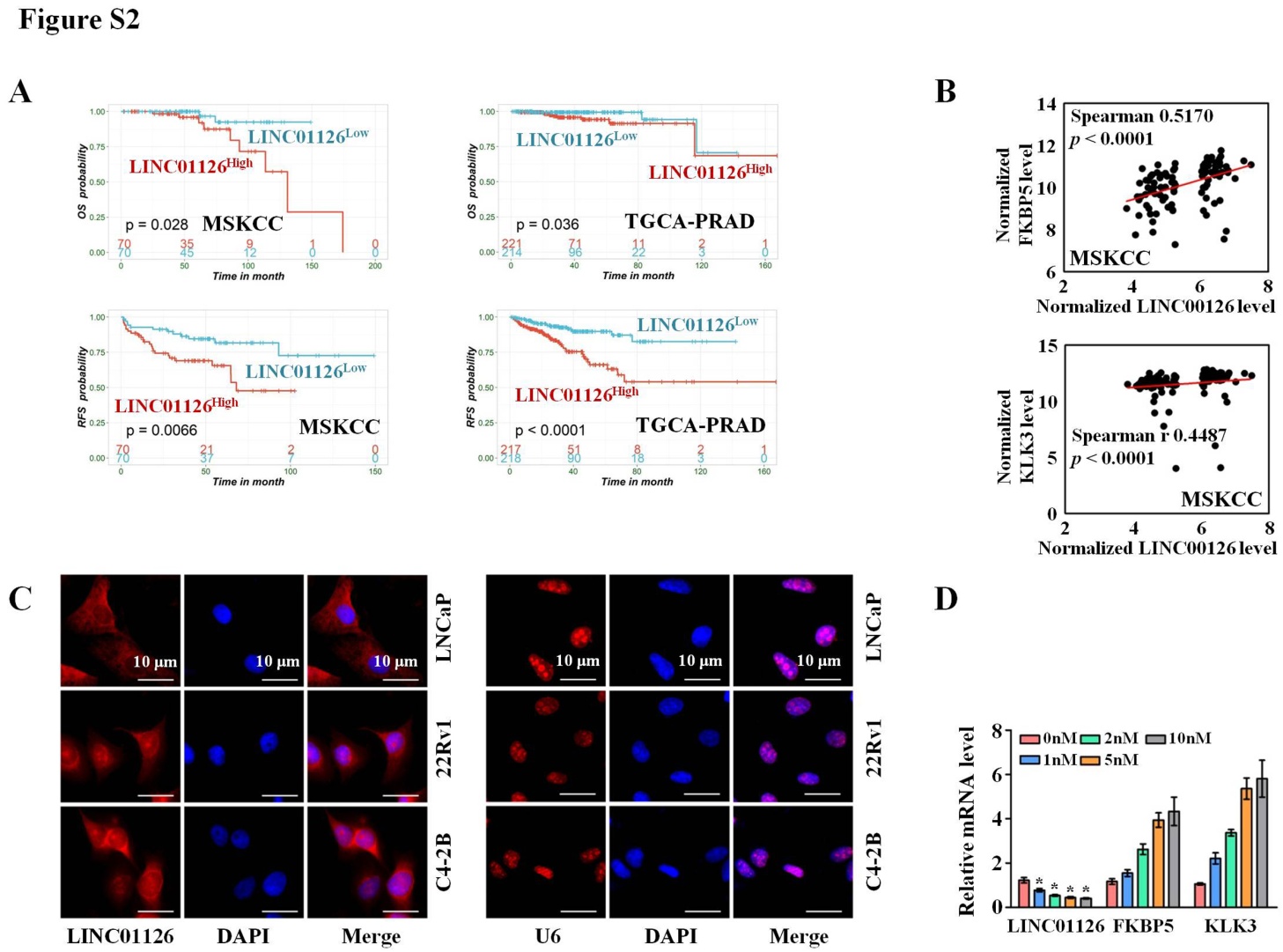
**

**Figure S2. (A)** Kaplan-Meier curves of the high-LINC01126 and low-LINC01126 groups. Patients in the high-LINC01126 group had a short overall survival time (*p* = 0.028 for MSKCC cohort and *p* = 0.036 for TGCA-PRAD cohort) and a short progression free survival time (*p*= 0.0066 for MSKCC cohort and *p* < 0.0001 for TGCA-PRAD cohort) relative to those in the low-LINC01126 group. **(B)** Spearman correlation analysis confirmed that LINC01126 expression in tumor tissues derived from MSKCC clinical prostate cancer cohort was positively correlated with the mRNA expression of KLK3 and FKBP5. **(C)** LINC01126 expression was detected by RNA fluorescent in situ hybridization (FISH) assay in LNCaP, C4-2B and 22RV1 expression. The expression of U6 was measured to serve as the reference. Scale bar 10 μm. **(D)** Effect of AR ligand dihydrotestosterone (DHT) for 24 hours on LINC01126 expression in LNCaP cells was determined by qPCR. Data between 0 nM group and another group were analyzed with unpaired Student’s t-test with *p < 0.05.

# Supplementary Figure 3

#
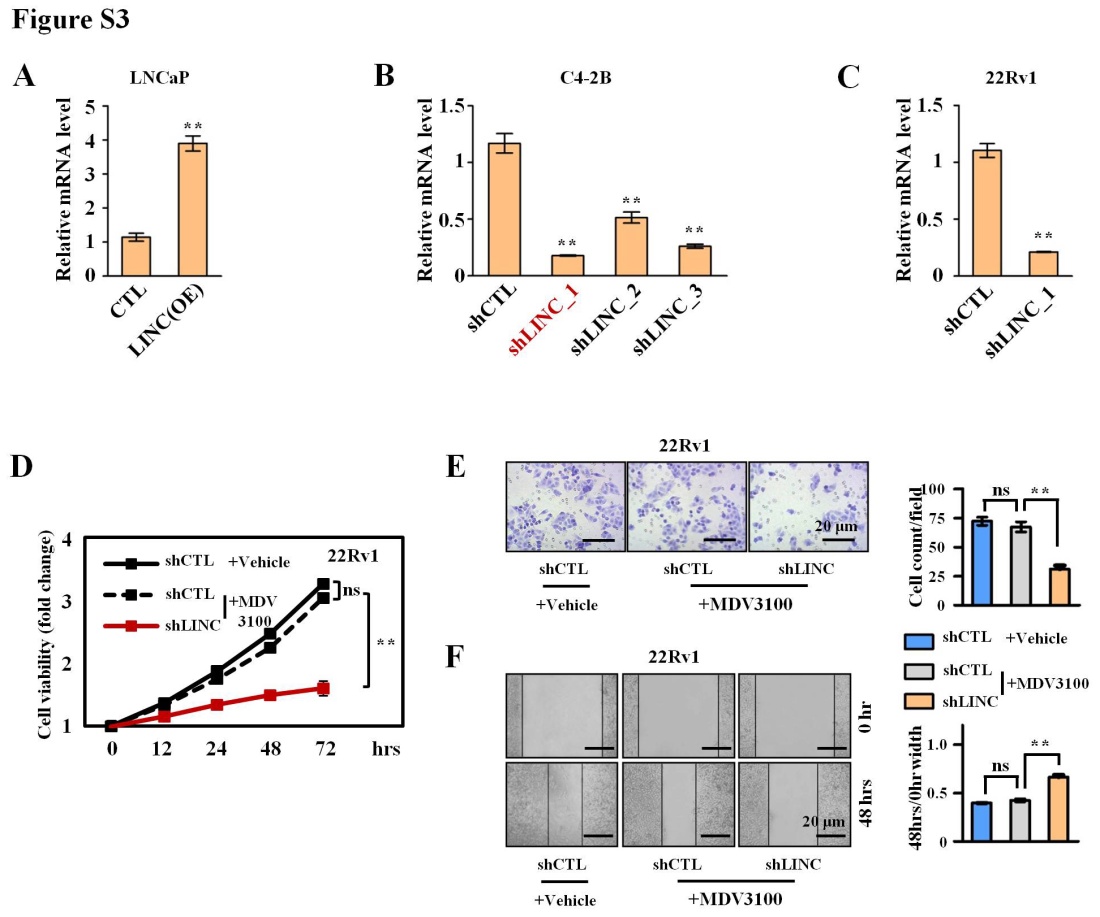


**Figure S3. (A)** The expression level of LINC01126 was measured by qPCR in LNCaP cells with or without (w/o) stable LINC01126 expression. **(B)** The expression level of LINC01126 was quantified by qPCR in C4-2B cells w/o stable LINC01126 knockdown. **(C)** The expression level of LINC01126 was measured by qPCR in 22Rv1 cells w/o stable LINC01126 knockdown. **(D)** CCK-8 assays for cell viability in the control and LINC01126-knockdown 22Rv1 cells without and with MDV3100 treatment (10 μM). Data are presented as the fold changes in cell viability during an observation period of up to 72 hours, and are representative of three replicate experiments (n = 3). Fold change on the day of cell seeding (hour 0) in each group was set as 1. **(E)** Representative images (left) and quantification of the invaded cells (right) derived from transwell assays of control and LINC01126-knockdown 22Rv1 cells without and with MDV3100 treatment (10 μM) for 48 hours (n = 3). Scale bar 20 μm. **(F)** Representative images (left) and quantification of cell migration (right) derived from wound healing assays of control and LINC01126-knockdwon 22Rv1 cells in the same context as **E** (n = 3). Scale bar 20 μm. CTL control, LINC(OE) LINC01126 overexpression, shCTL shControl, shLINC shLINC01126. ns, not significant, **p < 0.01.

**Supplementary Figure 4**

**
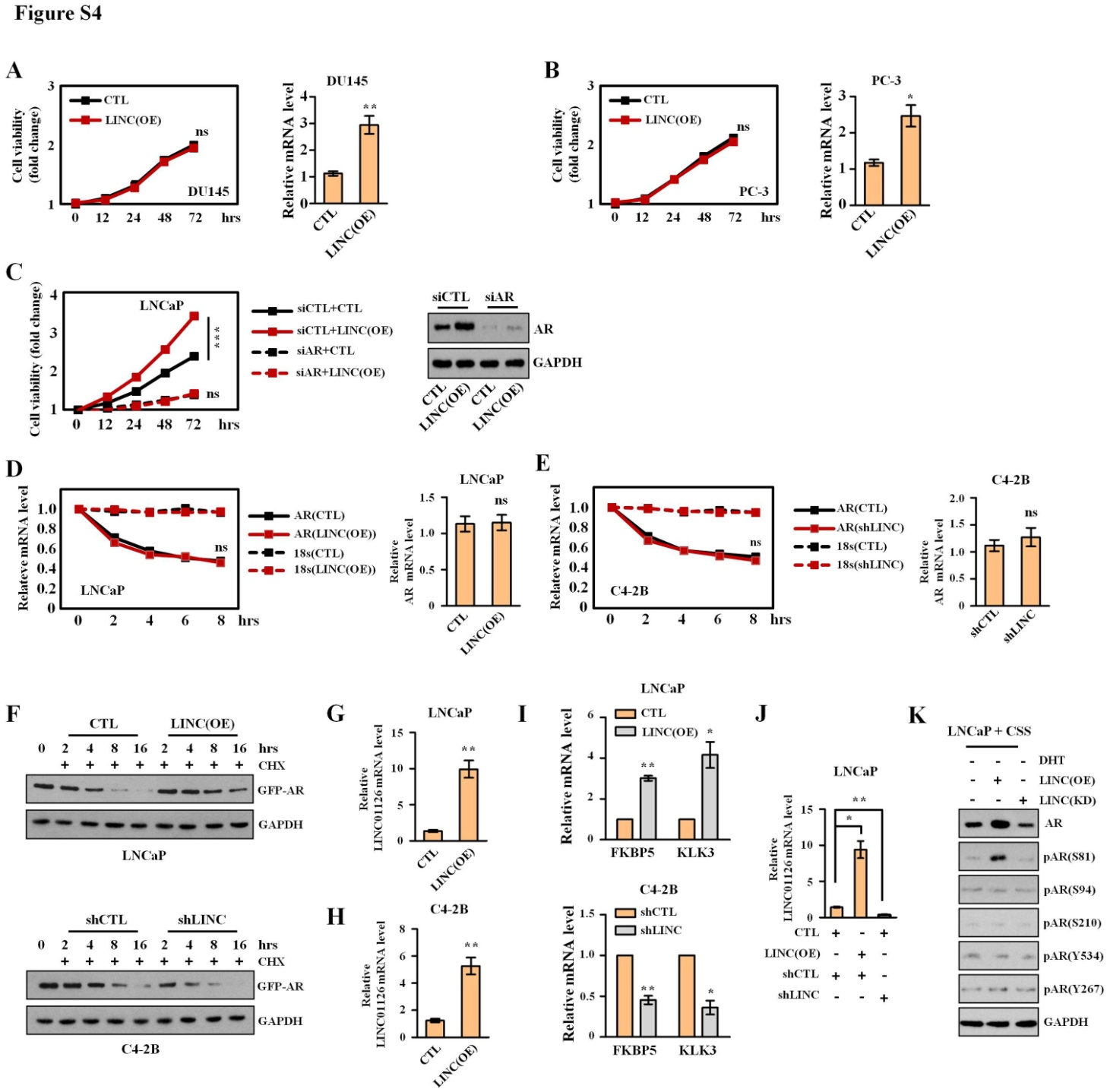
**

**Figure S4. (A-B)** CCK-8 cell viability assays (left) and LINC01126 level (right) in the control and LINC01126-stable overexpressing DU145 cells **(A)** and PC-3 cells **(B)**. **(C)** The control and LINC01126-stable overexpressing LNCaP cells were further treated with or without (w/o) siRNA pool targeting AR. CCK-8 cell viability assays (left) and the AR protein level of these cells (right) that was measured by immunoblotting. **(D)** The control and LINC01126-stable overexpressing LNCaP cells were treated with actinomycin D (5 μg/ml), and the expression level of AR and 18s was measured by qPCR for the indicated times (left). The mRNA expression level of AR was also examined in LNCaP cells w/o LINC01126 overexpression by qPCR (right). **(E)** The control and LINC01126-stable knockdown C4-2B cells were treated with actinomycin D (5 μg/ml), the expression level of AR and 18s was measured by qPCR for the indicated times (left). The mRNA expression level of AR was also examined in C4-2B cells w/o LINC01126 knockdown (right). **(F)** Cells were treated with CHX (10 ug/ml) for the indicated times to inhibit protein synthesis and to test for stability of exogenous AR protein with GFP tagged in LNCaP cells w/o LNC01126 overexpression (up) and in C4-2B cells w/o LNC01126 knockdown (down). **(G)** The expression level of LINC01126 in LNCaP cells w/o transient LINC01126 overexpression for 24 hours was measured by qPCR. **(H)** The expression level of LINC01126 in C4-2B cells w/o transient LINC01126 overexpression for 24 hours was measured by qPCR. **(I)** The expression level of FKBP5 and KLK3 was detected by qPCR in LNCaP cells w/o LINC01126 overexpression (up) and in C4-2B cells w/o LINC01126 knockdown (down). **(J)** The expression level of LINC01126 in LNCaP cells w/o transient LINC01126 overexpression and knockdown for 24 hours was measured by qPCR. **(K)** LNCaP cells w/o LINC01126 transient overexpression and knockdown were cultured in androgen-deprived medium for 24 hours. AR protein level and its [phosphorylation level](javascript:;) at several sites (S81, S94, S210, Y534, and Y267) were then measured by immunoblotting. Data between two groups were analyzed with unpaired Student’s t-test, ns not significant, *p < 0.05, **p < 0.01, and ***p < 0.001.

# Supplementary Figure 5

#
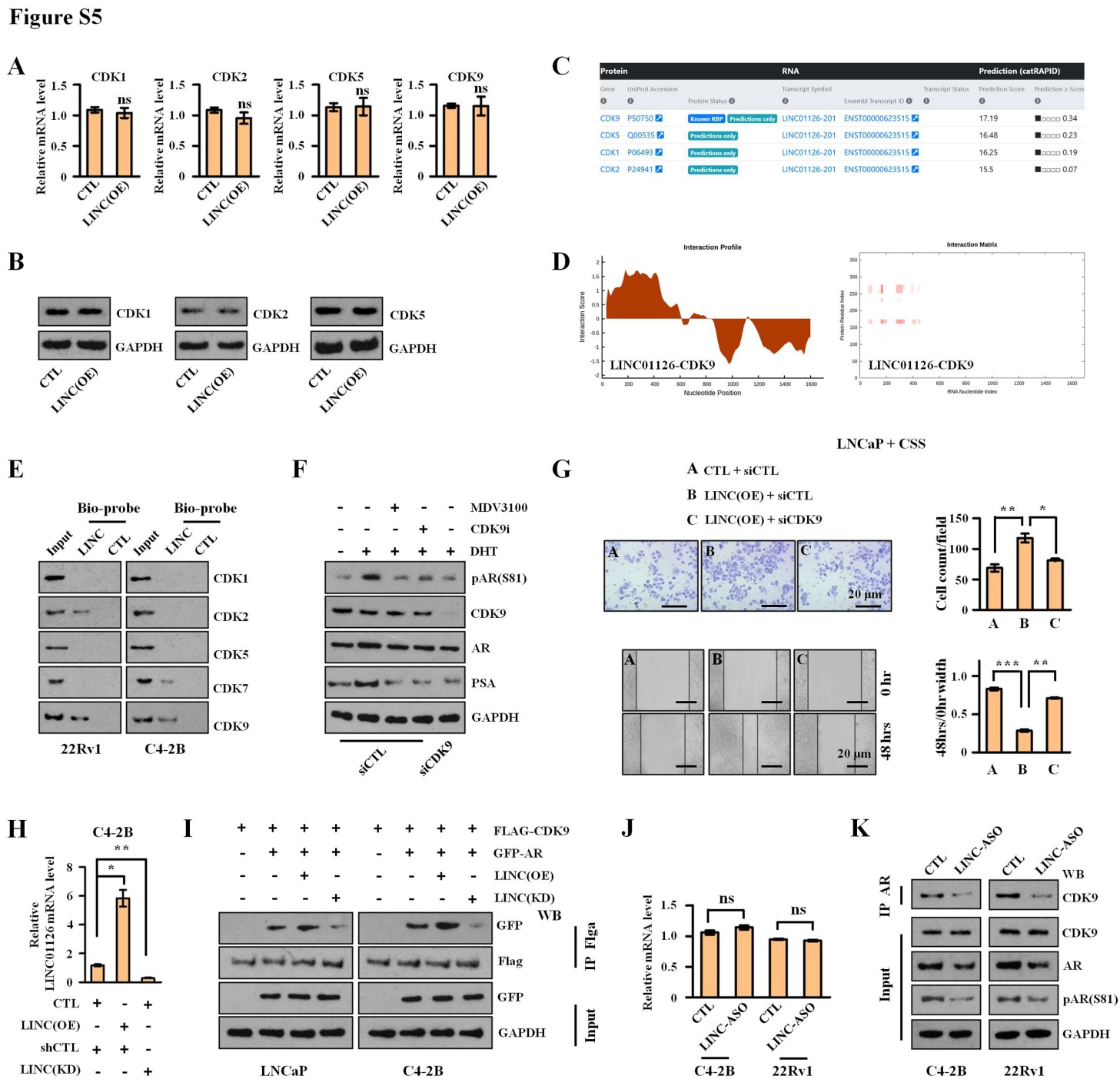


#
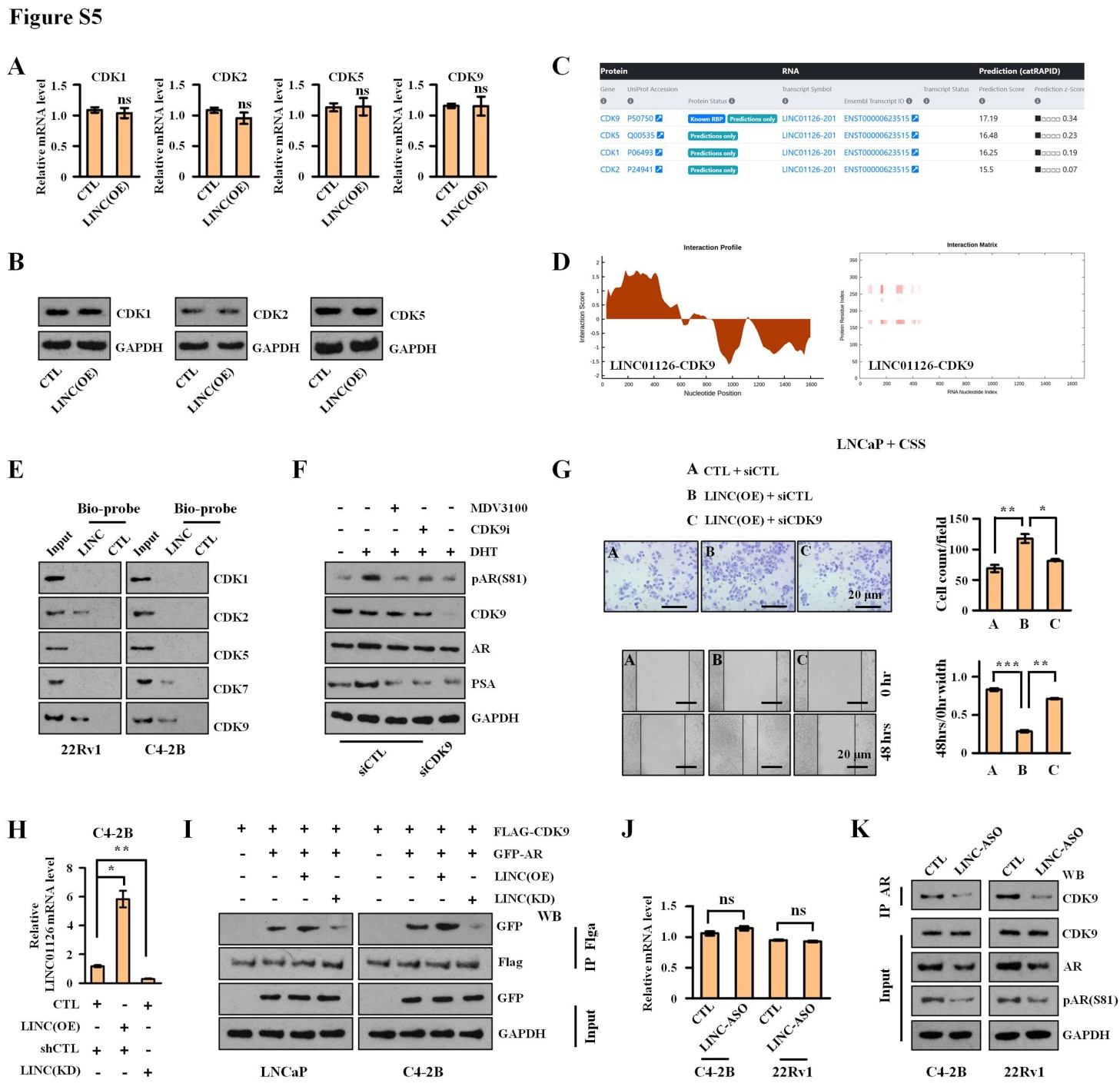


**Figure S5. (A)** The messenger RNA level of CDK1, CDK2, CDK5, and CDK9 was detected by qPCR in LNCaP cells with or without (w/o) LINC01126 overexpression. **(B)** The protein level of CDK1, CDK2, and CDK5 was detected by immunoblotting in LNCaP cells w/o LINC01126 overexpression. **(C)** CDK1, CDK2, CDK5, and CDK9 were predicted using catRAPID program to interact with LINC01126. **(D)** The catRAPID fragments module prediction of the interaction profile and matrix between CDK9 protein and LINC01126. **(E)** RNA pull-down assays in vitro with biotinylated LINC01126 or control probe followed by immunoblotting detection of the CDK proteins including CDK1, CDK2, CDK5, CDK7, and CDK9. **(F)** LNCaP cells w/o CDK9 knockdown were treated w/o DHT (10 nM), MDV3100 (10 μM), and CDK9 inhibitor BAY1251152 (10 ug/ml) for 24 hours. The levels of CDK9, AR, phosphorylated AR S81, and PSA were detected by immunoblotting. **(G)** Control and LINC01126-overexpression LNCaP cells were cultured in androgen-deprived medium w/o siRNA pool targeting CDK9 treatment. Representative images and quantification of the invaded cells derived from transwell assays (up) and representative images and quantification of cell migration derived from wound healing assays (down). Scale bar 20 μm. **(H)** The expression level of LINC01126 in C4-2B cells w/o transient LINC01126 overexpression and knockdown for 24 hours was measured by qPCR. **(I)** LNCaP (left) and C4-2B (right) cells w/o transient LINC01126 overexpression or knockdown were transfected with plasmid expressing CDK9-Flag together with AR-GFP plasmid. Flag-CDK9 was then immunoprecipitated with the anti-Flag antibody and blotted for GFP to examine the interaction of exogenous AR and CDK9. **(J)** C4-2B and 22Rv1 cells were treated w/o antisense oligos (ASO) targeting LINC01126 for 24 hours. Then the expression level of LNC01126 was measured by qPCR. **(K)** Immunoprecipitation and immunoblotting detection of AR/CDK9 interaction in C4-2B and 22RV1 cells w/o ASO targeting LINC01126. Data between two groups were analyzed with unpaired Student’s t-test, ns not significant, *p < 0.05, **p < 0.01, and ***p < 0.001.

# Supplementary Figure 6

#
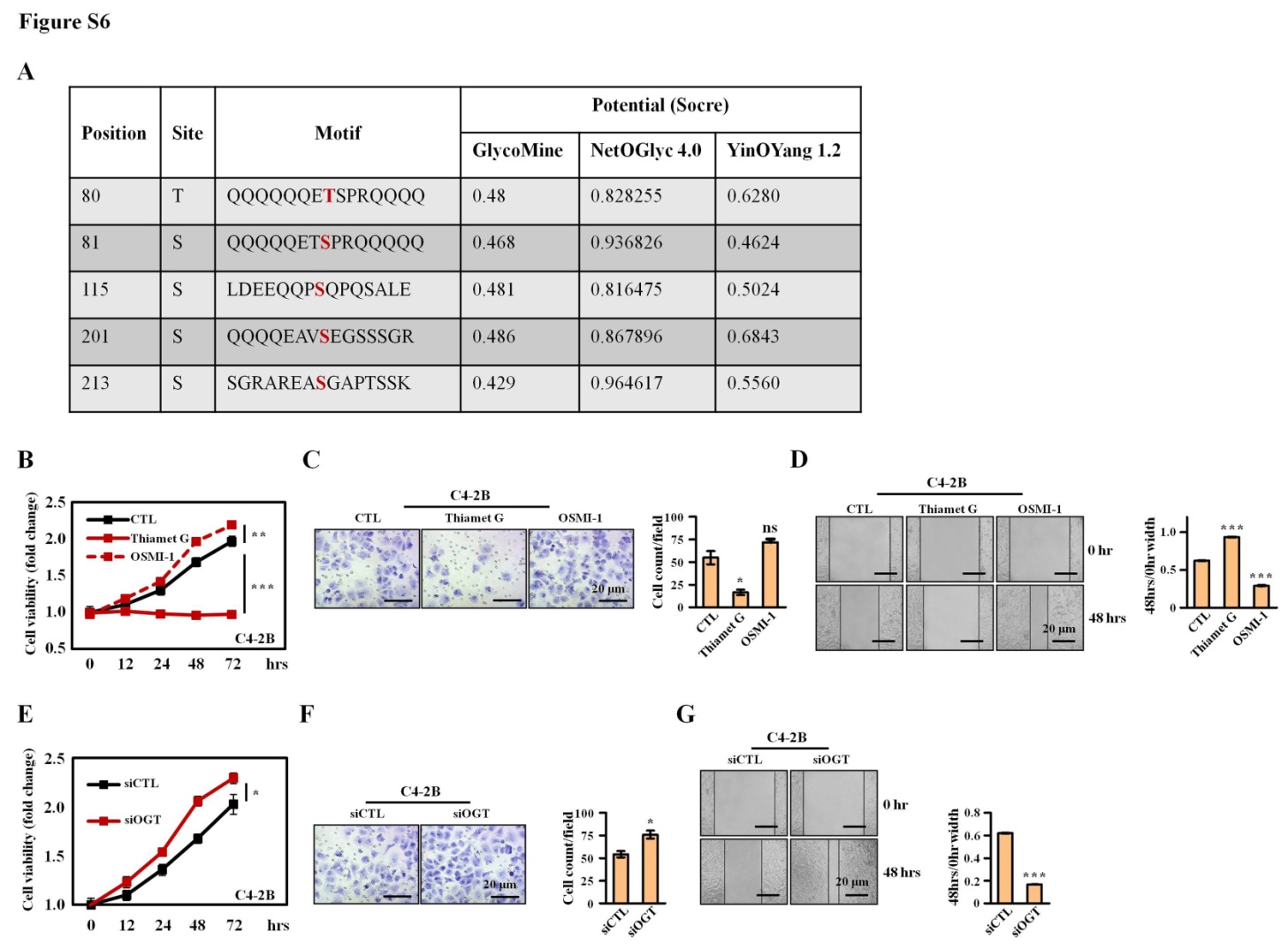


**Figure S6. (A)** The predicted O-GlcNAc sites on the AR protein using different online tools (YinOYang 1.2 Server, NetOGlyc 4.0 Server and G lycoMine Server). **(B-D)** C4-2B cells were treated with or without (w/o) Thiamet G (25 μM) or OSMI-1 (20 μM). The CCK-8 assay data were presented as fold changes in cell viability during an observation period of up to 72 hours (n=3). Fold change on the day of cell seeding (hour 0) in each group was set as 1 **(B)**. Representative images and quantification of the invaded cells derived from transwell assays **(C)** and representative images and quantification of cell migration derived from wound healing assays **(D)**. Scale bar 20 μm. Data between the CTL group and another group were analyzed with unpaired Student’s t-test. **(E-G)** C4-2B cells were treated w/o siRNA pool targeting OGT. The CCK-8 assay data were presented **(E).** Representative images and quantification of the invaded cells derived from transwell assays **(F)** and representative images and quantification of cell migration derived from wound healing assays **(G)**. Scale bar 20 μm. ns not significant, *p < 0.05, **p < 0.01, and ***p < 0.001.

# Supplementary Figure 7

#
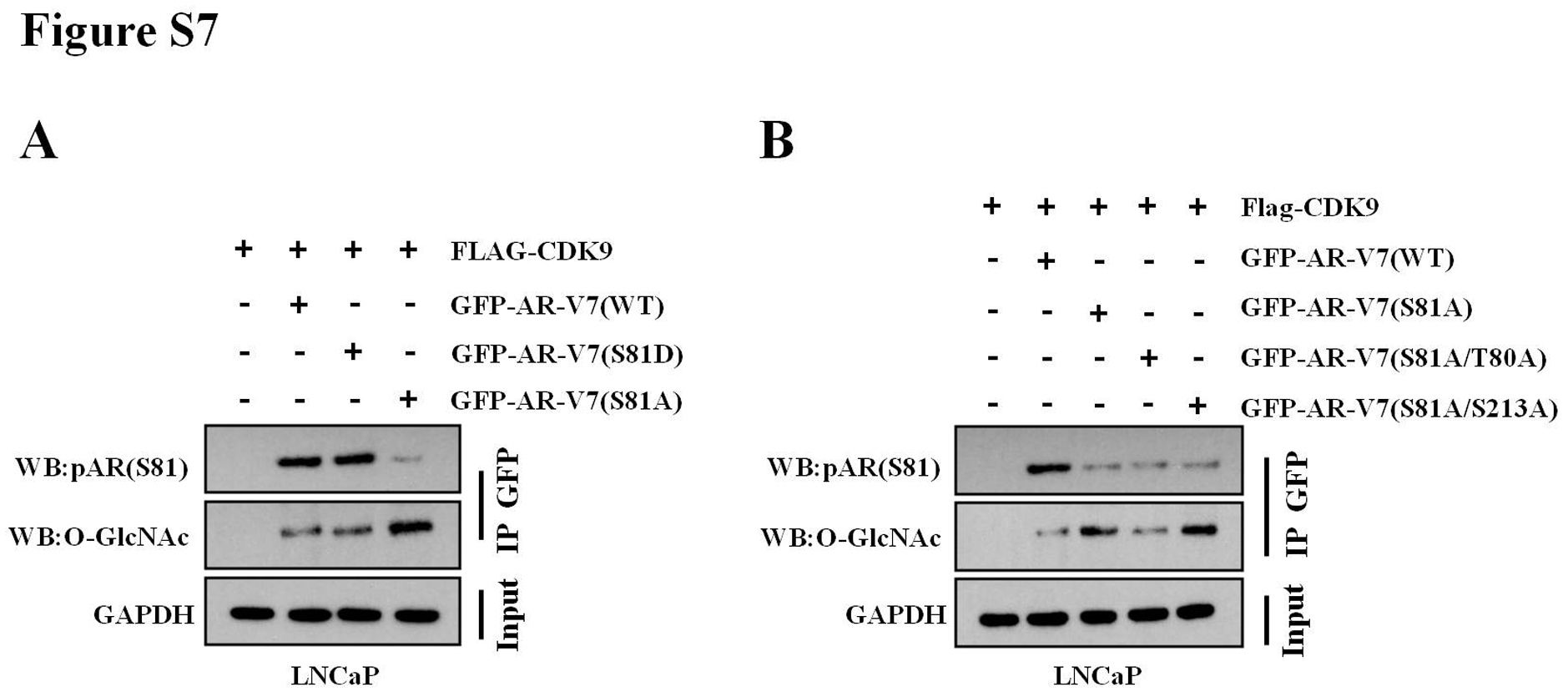


**Figure S7. (A-B)** LNCaP cells were transfected with plasmids expressing AR-V7-GFP (either WT or indicated mutant) together with CDK9-Flag plasmid. GFP-AR was immunoprecipitated with the anti-GFP antibody and blotted for O-GlcNAcylation and AR S81 phosphorylation.
